# A novel role of metal response element binding transcription factor 2 at the Hox gene cluster in the regulation of H3K27me3 by polycomb repressive complex 2

**DOI:** 10.18632/oncotarget.25505

**Published:** 2018-05-29

**Authors:** Abdul Aziz Khan, Seok-Jin Ham, Le Ngoc Yen, Haeng Lim Lee, Jounghyun Huh, Hyeongrin Jeon, Myoung Hee Kim, Tae-Young Roh

**Affiliations:** ^1^ Division of Integrative Biosciences and Biotechnology, Pohang University of Science and Technology (POSTECH), Pohang, Gyeongbuk 37673, Republic of Korea; ^2^ Department of Life Sciences, Pohang University of Science and Technology (POSTECH), Pohang, Gyeongbuk 37673, Republic of Korea; ^3^ Department of Anatomy, Embryology Laboratory, Yonsei University College of Medicine, Seoul 03722, Republic of Korea

**Keywords:** PRC2, MTF2, Hox genes, epigenetic silencing, differentiation

## Abstract

Polycomb repressive complex 2 (PRC2) is known to play an important role in the regulation of early embryonic development, differentiation, and cellular proliferation by introducing methyl groups onto lysine 27 of histone H3 (H3K27me3). PRC2 is tightly associated with silencing of Hox gene clusters and their sequential activation, leading to normal development and differentiation. To investigate epigenetic changes induced by PRC2 during differentiation, deposition of PRC2 components and levels of H3K27me3 were extensively examined using mouse F9 cells as a model system. Contrary to positive correlation between PRC2 deposition and H3K27me3 level, down-regulation of PRC2 components by shRNA and inhibition of EZH1/2 resulted in unexpected elevation of H3K27me3 level at the Hox gene cluster despite its global decrease. We found that metal response element binding transcriptional factor 2 (MTF2), one of sub-stoichiometric components of PRC2, was stably bound to Hox genes. Its binding capability was dependent on other core PRC2 components. A high level of H3K27me3 at Hox genes in *Suz12*-knock out cells was reversed by knockdown of *Mtf2*.This shows that MTF2 is necessary to consolidate PRC2-mediated histone methylation. Taken together, our results indicate that expression of Hox gene clusters during differentiation is strictly modulated by the activity of PRC2 secured by MTF2.

## INTRODUCTION

Epigenetic events control the expression of lineage-specific transcription factors by providing permissive or repressive environment in the nucleus. Such expression is associated with development and differentiation [1]. Genome-wide studies have shown that many genes are involved in embryonic development. Cell fate decisions can be silenced by polycomb group (PcG) proteins through H3K27me3 [2]. PcG proteins can restraint differentiation of pluripotent stem cells by rectifying histone methylation states (mono-, di-, and tri-methylated forms) and bring about repression of target genes. These proteins can induce and block some specific pathways responsible for the differentiation in a cell type-specific manner by controlling their target genes’ expression [3–5]. PcG proteins form a huge complex called polycomb repressive complex (PRC) that oversees transcription of target genes through epigenetic histone modifications. Generally, there are two major classes of PRC; PRC1 and PRC2. EZH2-containing PRC2 complex catalyzes deposition of H3K27me3 at target sites to be repressed while PRC1 introduces mono-ubiquitination of H2AK119 [6, 7]. Both PRC1 and PRC2 are involved in fine-tuning of transcriptional activity by altering chromatin states of developmentally regulated genes. During early embryonic development and ES cells differentiation, heterochromatin structure is progressively changed to euchromatin with gene expression [8].

The PRC2 complex can be found in two forms (PRC2- EZH2 and PRC2- EZH1) with two additional core components, SUZ12 and EED [9]. EZH2 is the major methyltransferase in the PRC2 complex. In the absence of EZH2, EZH1 might compensate for its function [10]. EZH2 is abundantly expressed in embryonic tissues and dividing cells whereas EZH1 is mostly found in non-dividing and differentiated cells [10–12]. EZH2 exhibits higher histone methyltransferase activity than EZH1 both *in vitro* and *in vivo* [10, 11]. Although the role of PRC2*-*EZH2 has been relatively well characterized [13, 14], the functional role of PRC2-EZH1 is not much explored. EZH2, a catalytic subunit of PRC2, is not sufficient for maintaining stem cell identity and pluripotency without forming a complete complex with other core PRC2 components like SUZ12 and EED [8, 10]. In the absence of SUZ12, the expression level of EZH2 is severely decreased, and thus the global levels of H3K27me3 diminish and cellular proliferation is attenuated [15, 16]. *Eed* knockout embryonic stem (ES) cells can retain the ability to differentiate into all three germ layers and form chimera. However, without SUZ12, ES cells cannot proceed to proper cellular differentiation along with defective gene expression essential for stem cell function [16, 17].

In *Drosophila*, PCL (polycomb-like) has been identified as an accessory component of PRC2 complex [18, 19]. Histone methyl transferase activity of PRC2 is enhanced in the presence of PCL [20]. MTF2 or PCL2, a mammalian orthologue of PCL, has the same function as PCL in *Drosophila*. MTF2 protein has a Tudor domain and two tandem PHD (plant homeodomain) zinc finger motifs that are associated with core PRC2 components. MTF is uniquely expressed in mouse ES cells and involved in early embryonic development and mostly enriched at polycomb target genes. MTF2 knockdown increased self-renewal activity of ES cells and blocked differentiation by decreasing H3K27me3 at specific targets [21].

Like ES cells, embryonal carcinoma (EC) cells have also been widely used as a model system for differentiation-related analysis as well as cancer stem cell studies. EC cells express majority of ES cell marker genes and can be easily grown in a short time by using limited resources. Retinoic acid (RA) treatment of EC cells induces differentiation into primitive endodermal cells by the displacement of PcG proteins and H3K27me3 on target genes [22–25]. Murine F9 cells are multipotent cell lines that can be maintained for a longer time without spontaneous differentiation [26, 27]. Similar to ES cells, F9 cells can also repress the transcription of exogenous retroviral DNAs by epigenetic mechanisms [28]. Despite efficient integration of viral sequences into the genome, F9 cells are resistant to expression of genes driven by viral promoters in undifferentiated state. However, differentiation may release this epigenetic repression and restore the transcription of viral sequences [29].

In this study, epigenetic changes were carefully examined during differentiation of F9 cells. Genome-wide distribution of H3K27me3 and binding profiles of PRC2 components were compared with gene expression, especially Hox cluster genes. Impacts of knockdown of individual core PRC2 components and EZH1/2-specific inhibitor were analyzed to understand the direct role of PRC2 in the management of H3K27me3 at Hox loci. Finally, MTF2 was identified as a key component in the regulation of transcription at Hox loci as well as in the accumulation of H3K27me3 in the absence of PRC2.

## RESULTS

### Differentiation of F9 cells to primitive endoderm

**Figure 1:**
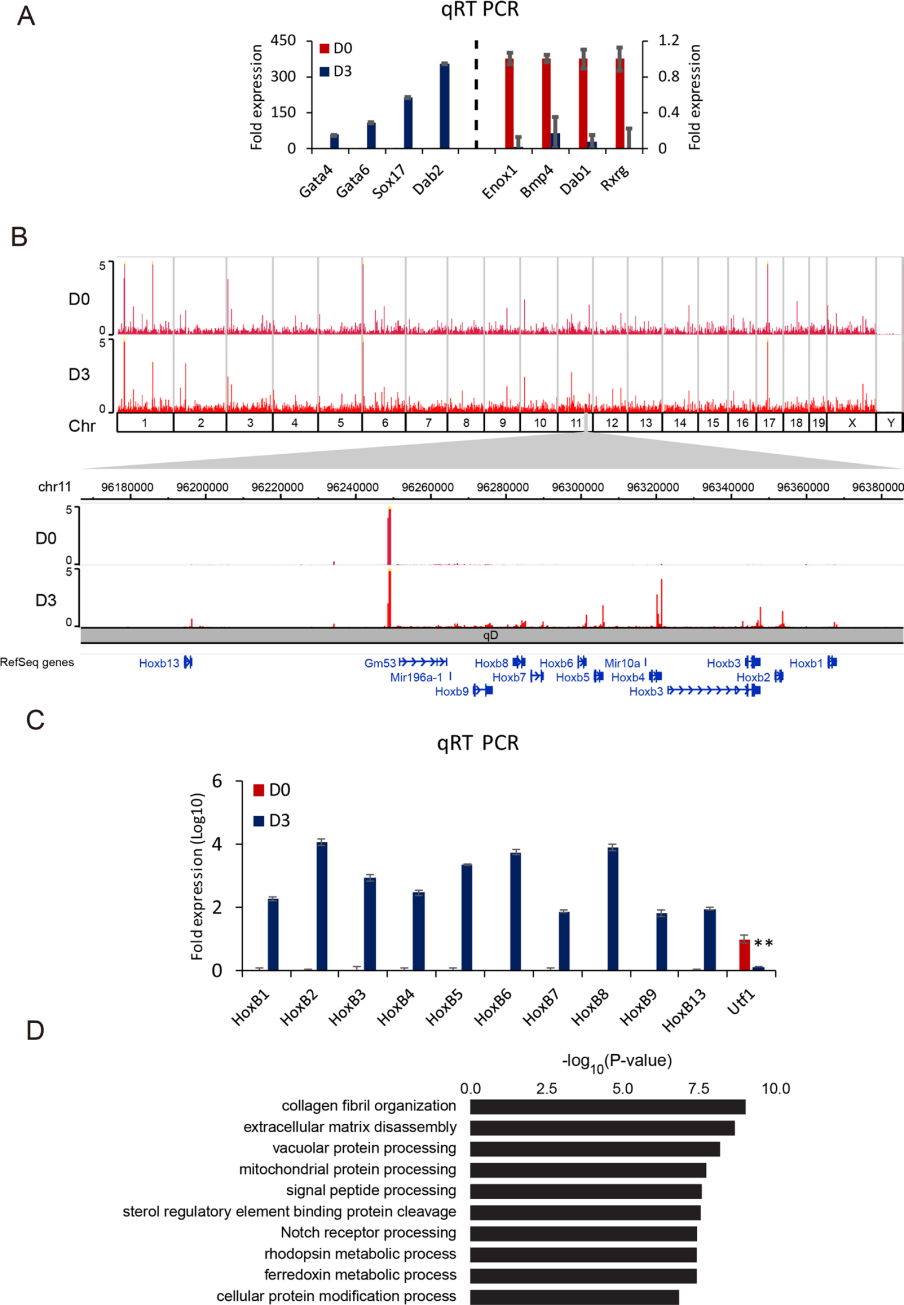
RA differentiates F9 cells to primitive endodermal cells (**A**) qRT PCR analysis for expression levels of primitive endodermal marker genes at D3 relative to those at D0 (left panel) and downregulation of genes at D3 compared to those at D0 (right panel). The y-axis represents fold expression level normalized by taking the expression of D0 as 1. Error bars represent mean ± SD of three biological replicates. ^*^P < 0.05, ^**^P < 0.01, ^***^P < 0.001 by Student’s two-tailed *t*-test. For all bars, *P* values were less than 0.001 unless otherwise specified. (**B**) An overall view of genomic expression at D0 and D3. The top panel shows entire genes on whole chromosomes. The bottom panel shows Hox B cluster genes. (**C**) Verification of relative expression changes of Hox B cluster genes in RA differentiating cells by qRT PCR. *Utf1* was used as a control. Error bars represent mean ± SD of three biological replicates. ^*^P < 0.05, ^**^P < 0.01, ^***^P < 0.001 by Student’s two-tailed *t*-test. For all bars, *P* values were less than 0.001 unless otherwise specified. (**D**) GO analysis of up-regulated genes at D3 compared to those at D0. Bars represent –log_10_ of *p*-values.

To explore transcriptional changes during differentiation, F9 cells were treated with RA for 3 days (D3) and compared with control (D0, F9 cells without RA treatment). The efficiency of differentiation was examined by qRT PCR using endodermal marker genes such as *Gata4*, *Gata6*, *Sox17,* and *Dab2* as primers. F9 cells were confirmed to be properly differentiated (Figure 1A). Representative decrease in the expression of genes such as *Enox1*, *Bmp4*, *Dab1,* and *Rxrg* was validated upon RA differentiation. Genome-wide expression profiles in F9 cells were analyzed by mRNA-Seq at D0 and D3. Chromosome-wide profiles and expression levels of Hox cluster genes are shown in Figure 1B. Noticeable changes at D3 were detected for Hox cluster genes (Hox B in Figure 1B, others in Supplementary Figure 1A–1C). The expression of Hox A cluster gene was also high while expression levels of Hox C and D cluster genes were relatively low (Supplementary Figure 1A–1C). The gene induction by RA treatment was validated by qRT PCR with Hox B cluster genes (Figure 1C). At D3, 1,753 genes were up-regulated while 944 genes were down-regulated with a threshold of absolute fold change > 2 and FDR < 10^–3^. During differentiation, the top 10 gene ontology (GO) terms were identified with high significance. Among them, genes involved in collagen fibril organization, extracellular matrix disassembly, and vacuolar protein processing were ranked in the top three GO terms (Figure 1D). These results suggest that RA treatment could differentiate F9 cells to primitive endoderm and highly induce the transcription of the Hox gene cluster.

### Enrichment profiles of EZH2 and H3K27me3 at Hox loci during differentiation

**Figure 2:**
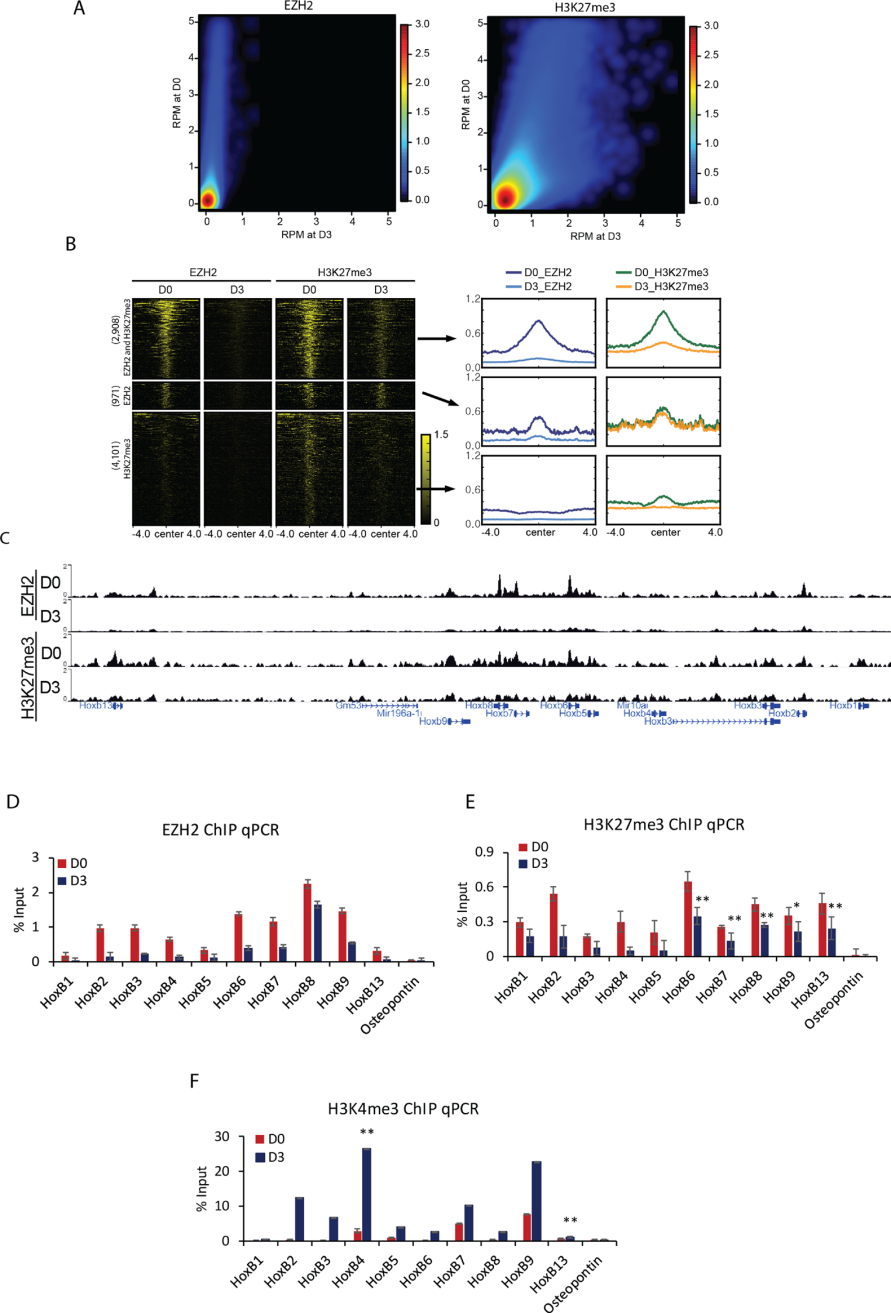
Displacement of H3K27me3 and PRC2 during cellular differentiation induced by RA (**A**) Colored scatterplots of bins with length of 1 kb occupied by EZH2 (left) and H3K27me3 (right) at D0 and D3. (**B**) Heatmaps of ChIP-Seq signals ± 4 kb from the center of regions enriched with EZH2 and H3K27me3 common regions, EZH2 specific regions, and H3K27me3 specific regions (left). Metagene plots depicts the average pattern of EZH2 and H3K27me3 (right). (**C**) A genomic view showing that both EZH2 and H3K27me3 were strongly decreased at D3 compared to those at D0. Verification of the effect of (**D**) EZH2 or (**E**) H3K27me3 displacement from Hox B cluster genes by ChIP qPCR. *Osteopontin* was used as a negative control. (**F**) Accumulation of H3K4me3 confirmed by ChIP qPCR in differentiating cells. ChIP results are shown relative to the input percentage. Error bars represent mean ± SD of three biological replicates. ^*^P < 0.05, ^**^P < 0.01, ^***^P < 0.001 by Student’s two-tailed *t*-test. For all bars, *P* values were less than 0.001 unless otherwise specified.

During differentiation, the relationship between the level of H3K27me3 and occupancy of PRC2 components was explored. A comparative analysis between D0 and D3 showed a decrease in the enrichment of EZH2 and H3K27me3 at D3. The degree of decrease in EZH2 was more substantial compare to that of H3K27me3 (Figure 2A). Next, EZH2 and H3K27me3 enriched regions showing decrease in enrichment at D3 were divided into three groups: 2,908 regions with both decrease of EZH2 and H3K27me3, 971 regions with decrease of EZH2 only, and 4,101 regions with decrease of H3K27me3 only (Figure 2B). Co-occupied regions with both decrease of EZH2 and H3K27me3 showed positive correlation in enrichment patterns in the whole genome level and at the localized peaks (Supplementary Figure 2A, 2B). EZH2 and H3K27me3 were relatively depleted at D3 compared to those at D0 (Supplementary Figure 2C).

Consistent with the fact that Hox B cluster genes were transcriptionally induced by RA, histone methyltransferase EZH2 enrichment was decreased and patterns of H3K27me3 were strongly depressed at Hox B loci (Figure 2C). The decrease in enrichment of EZH2 and H3K27me3 was confirmed by ChIP qPCR (Figure 2D, 2E). A similar reduction was observed for SUZ12 and EED at Hox B loci (Supplementary Figure 2D, 2E). Other Hox cluster genes such Hox A, C, and D also showed sharp decrease in the binding of EZH2 and introduction of H3K27me3 (Supplementary Figure 2F–2H). As expected, the level of active histone mark H3K4me3 was augmented at Hox B genes (Figure 2F). Genes occupied commonly by EZH2 and H3K27me3 were related to the following biological processes: pattern specification process, regionalization, cell fate commitment, and so on (Supplementary Figure 2I). However, these events were not detected in other chromatin regions occupied only by H3K27me3 (Supplementary Figure 2J). Taken together, these results suggest that Hox genes are repressed by PRC2 through H3K27me3 in undifferentiated cells and RA signal can induce the increase of H3K4me3 for transcriptional activation.

### Unusual accumulation of H3K27me3 at Hox loci by down-regulation of EZH2

**Figure 3:**
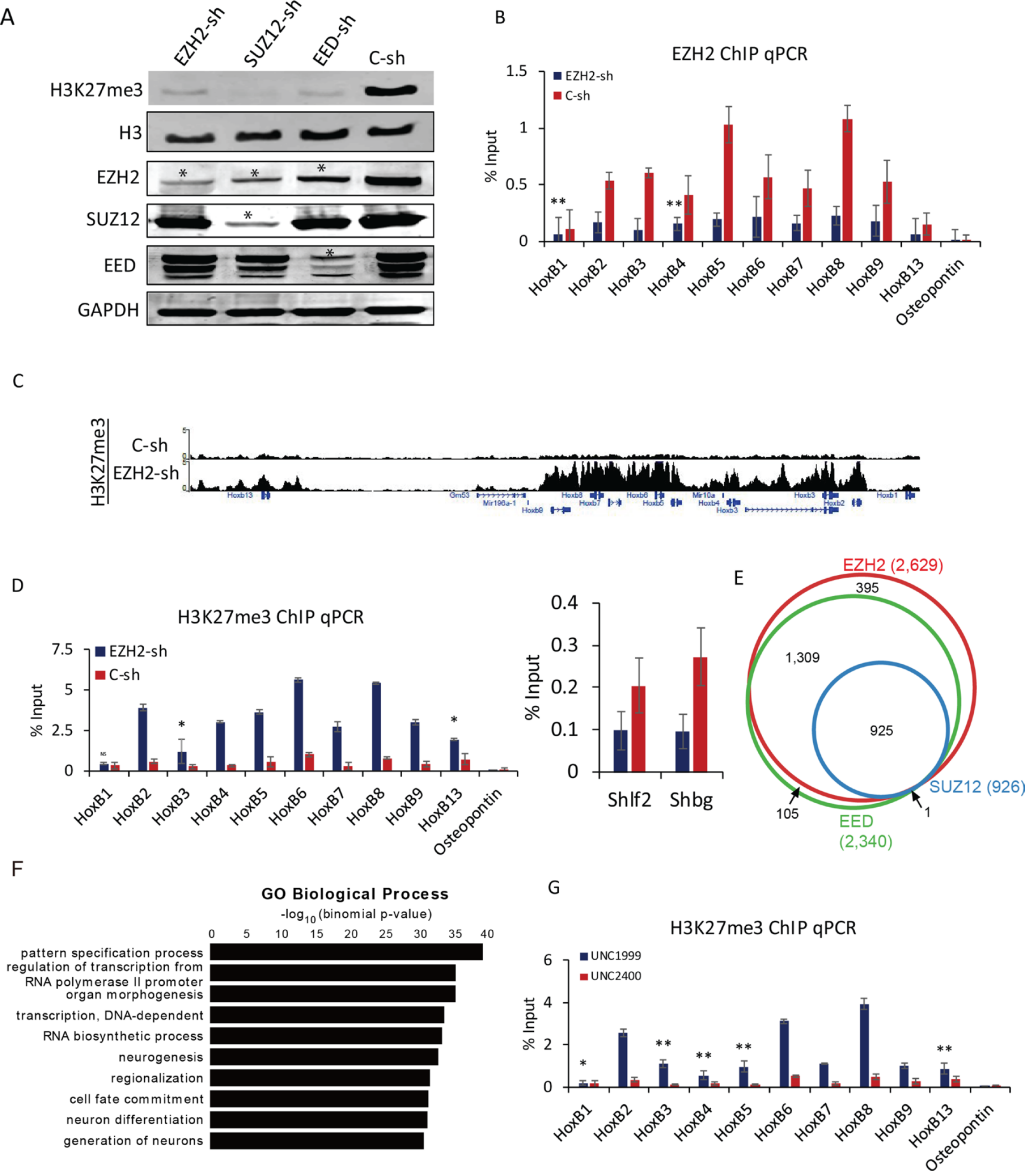
Depletion of Ezh2 induce H3K27me3 at Hox loci (**A**) Western blot analysis of cellular components after knockdown of *Ezh2, Suz12*, and *Eed*. H3K27me3 was strongly decreased. GAPDH and H3 were used as loading controls. Star (^*^) denotes decrease in protein expression. (**B**) ChIP qPCR of EZH2 binding to Hox B cluster genes in *Ezh2* knockdown. *Osteopontin* was used as a negative control. Error bars represent mean ± SD of three biological replicates. ^*^P < 0.05, ^**^P < 0.01, ^***^P < 0.001 by Student’s two-tailed *t*-test. For all bars, *P* values were less than 0.001 unless otherwise specified. (**C**) A genomic view of H3K27me3 with increased H3K27me3 at Hox B loci in *Ezh2* knockdown. (**D**) Verification with ChIP qPCR. *Osteopontin* was used as a negative control. *Shlf2* and *Shbg* were used as controls to show decreased H3K27me3 in *Ezh2* knockdown. Error bars represent mean ± SD of three biological replicates. ^*^P < 0.05, ^**^P < 0.01, ^***^P < 0.001 by Student’s two-tailed *t*-test. For all bars, *P* values were less than 0.001 unless otherwise specified. (**E**) A Venn diagram showing regions enriched with H3K27me3 upon knockdown of *Ezh2*, *Suz12*, and *Eed*. (**F**) GO analysis of regions with enrichment of H3K27me3 in all three conditions. A total of 925 common H3K27me3-enriched regions were used for GO analysis. Bars represent -log_10_ of *p*-values. (**G**) ChIP qPCR showing the increase in enrichment of H3K27me3 after inhibition of EZH1/2 by UNC1999. UNC2400 was used as control. Error bars represent mean ± SD of three biological replicates. ^*^P < 0.05, ^**^P < 0.01, ^***^P < 0.001 by Student’s two-tailed *t-*test. For all bars, *P* values were less than 0.001 unless otherwise specified.

PRC2 plays a critical role in the transcriptional regulation of Hox genes during differentiation [8]. To further investigate the functional role of PRC2 in F9 cells, individual core PRC2 components *Ezh2*, *Suz12,* and *Eed* were knocked down using short hairpin RNA (shRNA) (Supplementary Figure 3A). Although the global level of H3K27me3 was strongly decreased, each PRC2 component was not completely depleted (Figure 3A). The expression levels of all PRC2 components (EZH2, SUZ12, and EED) were repressed by individual shRNA. The expression of EZH2 was also decreased by knockdown of either *Suz12* or *Eed* (Figure 3A). A substantial decrease in PRC2 enrichment at Hox B loci was confirmed by ChIP qPCR when *Ezh2*, *Suz12,* or *Eed* was independently knocked down (Figure 3B, Supplementary Figure 3B, 3C). Contrast to the down-regulation of H3K27me3 level corresponding to decreased EZH2 binding at Hox loci during differentiation (Figure 2D), the deposition of H3K27me3 was dramatically elevated even with partial depletion of EZH2 (Figure 3C). These changes in Hox B genes were further confirmed by qPCR (Figure 3D). The same effect was also observed in *Suz12* or *Eed* knockdown experiments (Supplementary Figure 3D–3F). Other Hox A, C, and D cluster genes also conceded an increase of H3K27me3 with depletion of each PRC2 component (Supplementary Figure 3G–3I). Regions significantly enriched with H3K27me3 in F9 cells treated with shRNA for *Ezh2*, *Suz12*, or *Eed* (over 2-fold increase compared to control shRNA) were found to be as 2,629, 926, and 2,340, respectively. Most of them were overlapped (Figure 3E). A total of 925 common H3K27me3-enriched regions were subjected to GO analysis. Genes maintaining high levels of H3K27me3 by perturbation of PRC2 complex were primarily categorized into biological processes such as pattern specification process, regulation of transcription from RNA polymerase II promoter, and organ morphogenesis (Figure 3F). The unexpected accumulation of H3K27me3 at Hox loci might be due to selective histone methylation by some residual PRC2 complex or the action of EZH1 known to complement EZH2 [10]. When treating F9 cells with UNC 1999, an EZH1/2 inhibitor, for 96 h, global level of H3K27me3 was strongly decreased in a dose-dependent manner. It was almost completely abolished by UNC 1999 at a concentration of 2 µM compared with UNC 2400, a close analog of UNC 1999 as a negative control (Supplementary Figure 3J). The strong decrease in global H3K27me3 level was due to the inhibition of EZH1/2 activity by treating UNC 1999. However, the level of H3K27me3 showed a significant increase at all Hox B cluster genes, indicating that EZH1 and/or EZH2 might not be absolutely needed for introducing methylation at histone H3 lysine 27 (Figure 3G). Moreover, the reduction in any of core PRC2 components could lead to the decrease of PRC2 binding to target sites and give rise to an unexpected hike of H3K27me3 (Supplementary Figure 3D, 3G–3I, 3K–3M, Figure 3C). Taken together, these observations reveal a novel regulatory role of PRC2 in the maintenance of a specific level of H3K27me3 at Hox loci.

### Hox cluster expression is not affected by PRC2 interference

**Figure 4:**
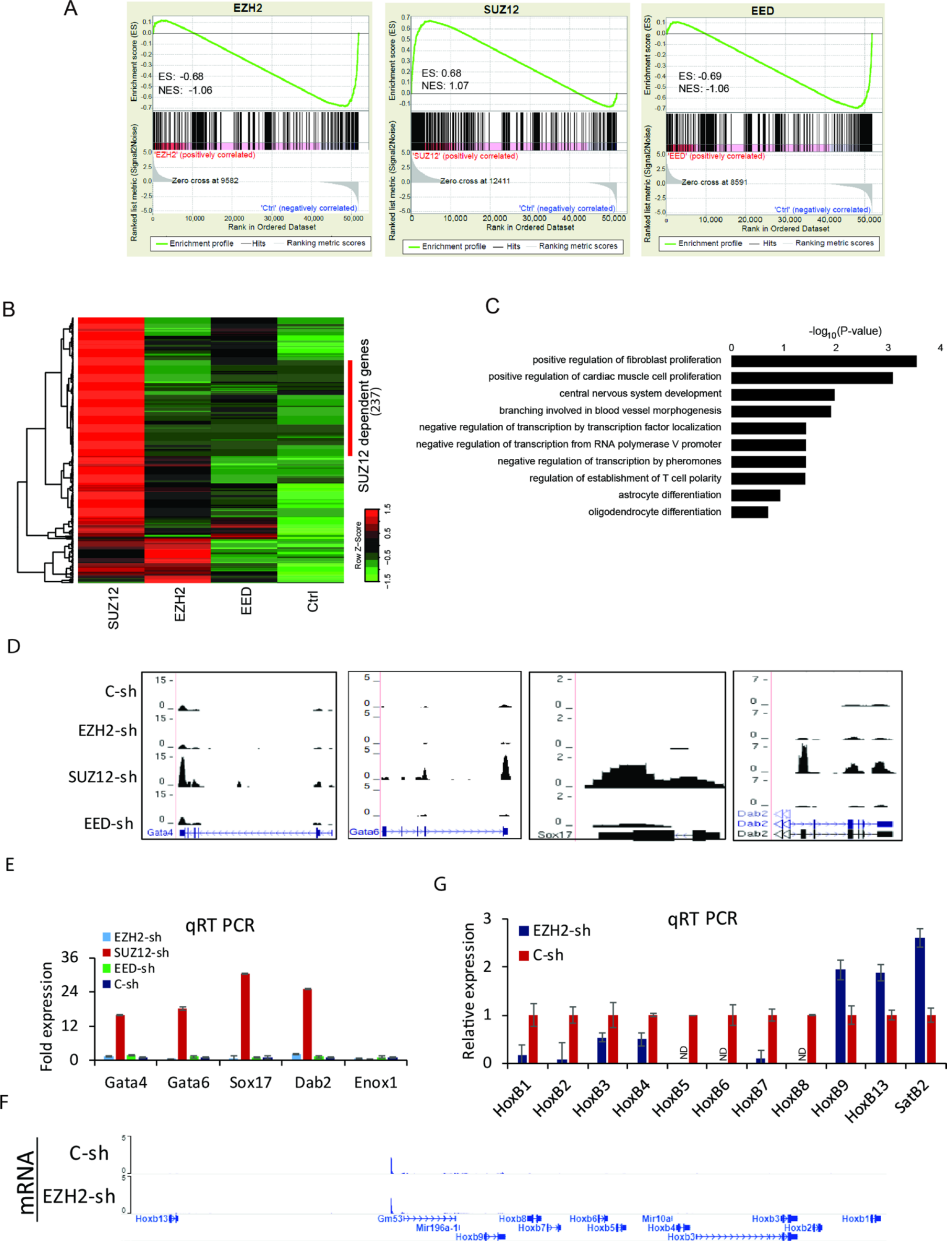
Transcriptome alterations in PRC2 depleted cells (**A**) GSEA for overall expression changes of the top 500 genes with H3K27me3 enhancement upon knockdown of *Ezh2* (left), *Suz12* (middle), and *Eed* (right). ES: enrichment score; NES: normalized enrichment score. (**B**) Heatmaps displaying the expression level of up-regulated genes at each knockdown compared to the control. (**C**) GO analysis for up-regulated genes specific to *Suz12* knockdown. Bars represent -log_10_ of *p*-values. (**D**) Genomic views of primitive endodermal marker genes. (**E**) Verification with qRT PCR for endodermal marker genes. Error bars represent mean ± SD of three biological replicates. ^*^P < 0.05, ^**^P < 0.01, ^***^P < 0.001 by Student’s two-tailed *t*-test. For all bars, *P* values were less than 0.001 unless otherwise specified. (**F**) A genomic view of Hox B loci showing no expression in *Ezh2* knockdown cells compare to control. (**G**) Verification with qRT PCR for Hox B cluster genes. *SatB2* was used as a control. Error bars represent mean ± SD of three biological replicates. ND: Not Detected.

To investigate the role of PRC2 in the transcriptional control at D0, gene expression profiles of *Ezh2*, *Suz12,* and *Eed* knockdown cells were analyzed by RNA-Seq. Gene Set Enrichment Analysis (GSEA) was performed using top 500 genes where H3K27me3 was increased upon PRC2 knockdown (Figure 4A). Expression changes of genes with elevated H3K27me3 upon PRC2 knockdown were negatively correlated with down-regulation of *Ezh2* (Enrichment score, ES= −0.68) or *Eed* (ES= −0.69). However, the enrichment score upon *Suz12* knockdown was positive (ES = 0.68). Despite high enrichment of H3K27me3, many genes displayed high expression in *Suz12*-KO cells. A total of 688 up-regulated genes compared to control at each knockdown (169 for *Ezh2*, 607 for *Suz12*, and 107 for *Eed*) were identified with a threshold of expression fold change > 2 and FDR < 10^–3^. Hierarchical clustering with relative distance less than 0.2 could classify 237 genes dependent on *Suz12* (Figure 4B). Their major functions are involved in positive regulation of fibroblast proliferation, positive regulation of cardiac muscle cell proliferation, central nervous system development, and so on (Figure 4C). The expression of primitive endodermal marker genes was only activated in *Suz12* knocked-down cells. Accordingly, their transcription levels were confirmed by RNA-Seq and qRT PCR (Figure 4D and 4E). The reduction of any PRC2 component further decreased the expression of Hox B cluster genes (Figure 4F, 4G, Supplementary Figure 4A, 4B). Similarly, the expression of Hox B genes during differentiation was reduced in knocked-down cells (Supplementary Figure 4C). Like Hox B cluster genes, Hox A, C, and D cluster genes were not increased in PRC2 depleted cells either (Supplementary Figure 4D–4F). The removal of core PRC2 components induced the impairment in the transcriptional activation of Hox genes. Although *Suz12* knockdown resulted in the activation of primitive endodermal marker genes, it was not related to the expression of Hox genes.

### Novel regulatory role of MTF2 in the absence of SUZ12

**Figure 5:**
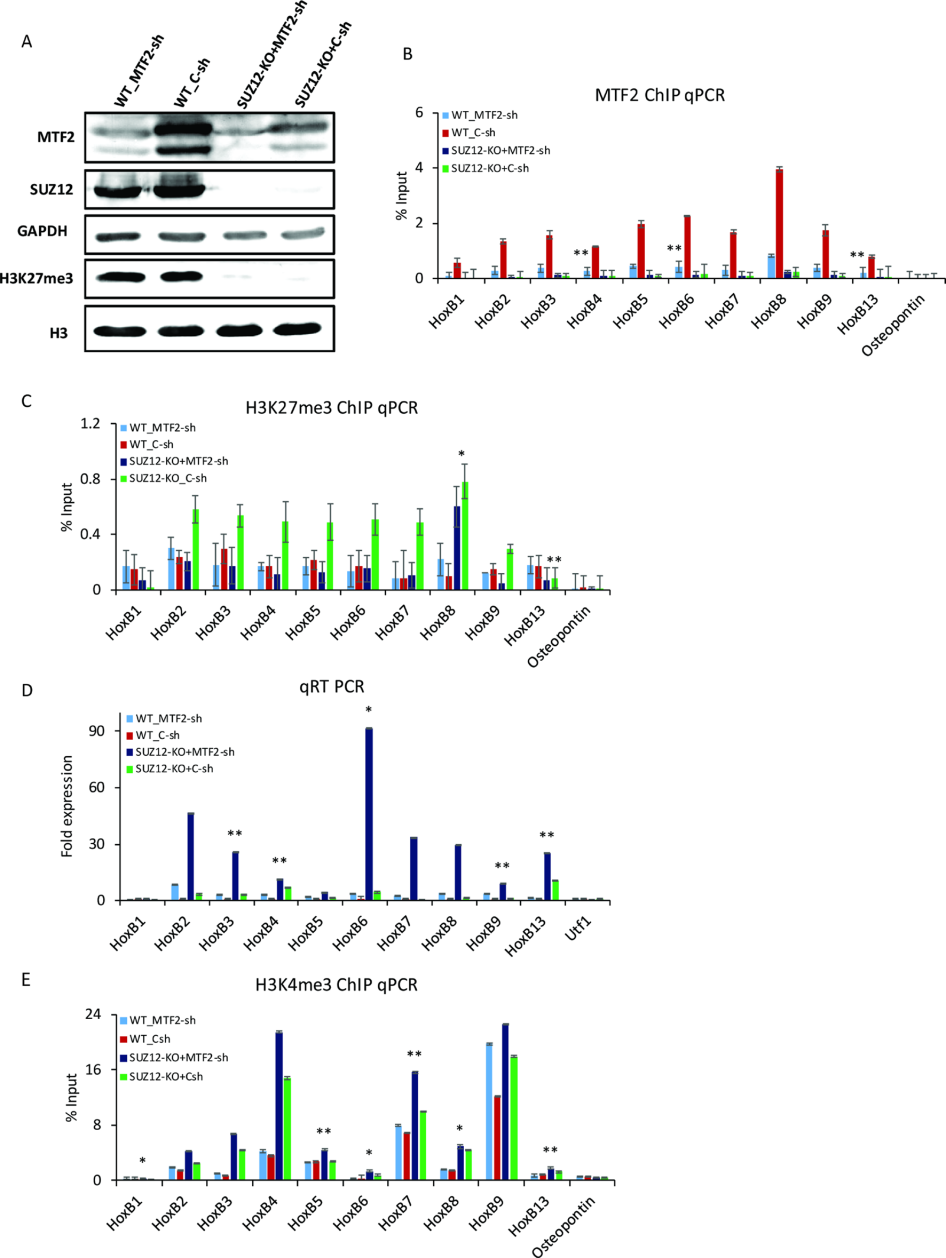
MTF2 regulates H3K27me3 at Hox B loci in the absence of SUZ12 (**A**) Western blot assay after the knockdown of *Mtf2* in WT and *Suz12*-KO. H3K27me3 did not change with knockdown of *Mtf2*, but disappeared with *Suz12* knockout. GAPDH and H3 were used as loading controls. ChIP qPCR for (**B**) MTF2 and (**C**) H3K27me3 at Hox B loci upon knockdown of *Mtf2* in WT and *Suz12*-KO. *Osteopontin* was used as a negative control. H3K27me3 was significantly decreased in *Suz12* and *Mtf2* double depletion compared to that in *Suz12*-KO. (**D**) qRT PCR for the expression of Hox B cluster genes. (**E**) ChIP qPCR for H3K4me3 of Hox B cluster genes. Error bars represent mean ± SD of three biological replicates. ^*^P < 0.05, ^**^P < 0.01, ^***^P < 0.001 by Student’s two-tailed *t*-test. For all bars, *P* values were less than 0.001 unless otherwise specified.

MTF2/ PCL2, an auxiliary component of the PRC2 complex, can enhance histone methyltransferase activity of PRC2 and repress the transcription of Hox genes [20, 30]. To examine the potential role of MTF2 in the regulation of H3K27me3 and Hox genes’ expression in D0 F9 cells, *Mtf2* was down-regulated by shRNA in wild-type cells (WT) and *Suz12* knockout cells (*Suz12*-KO). The expression of MTF2 was partially reduced in *Suz12*-KO. The effect of *Mtf2* shRNA was similar in both WT and *Suz12*-KO (Figure 5A). Protein level of SUZ12 was not changed regardless of MTF2 expression. In contrast, mRNA level of *Mtf2* in *Suz12*-KO was comparable to that in WT (Supplementary Figure 5A). The binding of MTF2 to Hox B genes was significantly decreased after *Mtf2* knockdown (Figure 5B). Furthermore, the recruitment of MTF2 to Hox B loci was strongly decreased in *Suz12*-KO, implying that MTF2 should be recruited to an intact PRC2 complex depending on SUZ12 (Figure 5B). Subsequently, the binding of SUZ12 was strongly curtailed in *Suz12*-KO as expected. However, it was also significantly decreased with the reduction of MTF2 at Hox B genes (Supplementary Figure 5B). The global level of H3K27me3 was completely diminished in *Suz12*-KO, but not changed with MTF2 depletion (Figure 5A). Compared to WT, the level of H3K27me3 at Hox B loci was enhanced in *Suz12*-KO (green bar), but not changed in *Mtf2* knockdown cells (light blue vs. red) (Figure 5C). Surprisingly, knockdown of *Mtf2* could counteract the effect of depletion of *Suz12* (dark blue vs. green). Moreover, along with restoration of H3K27me3 levels in WT, Hox B cluster genes showed high transcriptional induction in *Suz12*-KO+*Mtf2*-sh (Figure 5D). Subsequently, high levels of H3K4me3 were also confirmed in *Suz12*-KO+*Mtf2*-sh (Figure 5E). Although eminent H3K4me3 was maintained in *Suz12*-KO, it seemed to be insufficient to activate Hox B cluster genes in the presence of MTF2. Collectively, these observations suggest that MTF2 could indirectly control the enrichment of H3K27me3 in the absence of PRC2.

## DISCUSSION

In this study, F9 cells were differentiated to primitive endodermal cells by RA treatment and induced the expression of most Hox cluster genes. The activation of Hox genes was accompanied by reduced binding of core PRC2 components EZH2, SUZ12, and EED along with repressive histone marker H3K27me3. Depending on enrichment patterns of EZH2 and H3K27me3 at D0 and D3, genomic regions could be divided into three groups (decrease of both EZH2 and H3K27me3, decrease of EZH2 only, and decrease of H3K27me3 only). This observation suggests that the installment of H3K27me3 is not solely dependent on EZH2. Some unknown factors may contribute to the assembly of H3K27me3 at localized regions. Hox genes were included in the first group which showed strong decrease in the enrichment of PRC2 and H3K27me3 at D3 (Figure 2C, Supplementary Figure 2D–2H). Due to the substantial decrease in the binding of PRC2 and H3K27me3 at Hox loci, it was irrational to perform knockdown experiments in D3 cells. To investigate the deposition of H3K27me3 at Hox loci in D0 cells, we knocked down the expression of core PRC2 components *Ezh2*, *Suz12*, and *Eed* by specific shRNA independently. The global level of H3K27me3 was strongly decreased by the reduction of all core components. Downregulation of SUZ12 and EED reduced the expression of EZH2. However, knockdown of *Ezh2* was unable to decrease the expression of SUZ12 or EED. This result is consistent with previous observations showing that the stability of EZH2 protein is dependent on SUZ12 and EED [31, 32]. SUZ12 interacts and stabilizes EZH2 by its VEFS (VRN2, EMF2, FIS2 and Su(z)12) domain present at its C -terminal region by preventing proteasome-dependent degradation without affecting the expression of EED [15, 33]. The expression of EED was not affected by the reduction of EZH2 or SUZ12 in F9 cells. In the absence of EZH2 or SUZ12, PRC1 components BMI or RING1B may increase the stability of EED as both PRC1 components compete with EZH2 for EED binding. At higher concentration, they can even abrogate the enzymatic activity of PRC2 [32]. PRC2 also contains many sub-stoichiometric components. These auxiliary components of PRC2 may also play some roles in the stability of the individual core members. For example, expression levels of SUZ12 protein and mRNA are altered with knockdown and overexpression of Pcl3 (Polycomb-like 3) protein without affecting the stability of EZH2 or EED [34].

ChIP-Seq results showed a strong accumulation of H3K27me3 at Hox loci in PRC2 knockdown cells. Such increase in the enrichment of H3K27me3 was unexpected because not only global level of H3K27me3 was strongly reduced upon PRC2 knockdown, but also the binding of core components was significantly decreased by treatment with shRNA. Genes showing elevated levels of H3K27me3 were involved developmental processes such as organ morphogenesis, neurogenesis, cell fate commitment, and neuronal differentiation. Previous studies have reported that *Ezh1* can directly compensate the role of *Ezh2* in its absence by making a different complex called PRC2-EZH1 which contributes to localized level of H3K27me3 [10, 11]. To assess the potential role of EZH1 in the accumulation of H3K27me3 at Hox loci, F9 cells were treated with EZH1/2-specific inhibitor. The level of H3K27me3 was globally decreased with inhibition of EZH1/2. However, it is gene-specifically increased, especially at the Hox cluster genes. It is quite comparable to the accumulation of H3K27me3 at the same loci upon PRC2 knockdown. This observation ruled out the potential involvement of EZH1 in the absence of EZH2. De-repression of PRC2 target genes has been demonstrated in *Eed* ^*-/-*^ ESCs and *Suz12*-KO cells [3, 16, 17, 35], although other groups have claimed that KO of PRC2 does not lead to significant transcriptional changes in mESCs [36]. It seems that PRC2 can repress the expression of target genes in a cell type-specific manner. Functional reduction of PRC2 in F9 cells was unable to induce the expression of Hox genes. In contrast with *Ezh2* and *Eed*, knockdown of *Suz12* transcriptionally induced some genes, even in the presence of high level of H3K27me3. This observation was quite unexpected. However, the same outcome has been observed previously. It has been concluded that H3K27me3 cannot inhibit the transcription of genes even in the presence of PcG proteins [2, 16]. SUZ12 is the major PRC2 member that can sustain the stability of PRC2. As mentioned earlier, the loss of SUZ12 can decrease the expression of EZH2. Thus, we hypothesized that the same might happen for other components. They might facilitate the expression of PRC2 target genes. Knockout of Suz12 partially decreased the global level of MTF2. We could not exclude the possibility that reduced MTF2 might have a regulatory role in the activation of target genes. It is also possible that SUZ12 protein can physically block the recruitment of transcription machinery. Thus, the reduction of SUZ12 might pave the way for transcriptional induction. Although the reduction of SUZ12 induced the expression of some genes, it was unable to induce the expression of Hox genes. Our observations are completely opposite to the classical concept that PRC2 is the major repressive complex in the regulation of Hox gene transcription through H3K27me3 [37, 38].

By combining with sub-stoichiometric components, PRC2 can form two different complexes: PRC2-JARID and PRC2-MTF2. In *Jarid2* knockdown cells, core components of PRC2 are recruited and the increase of H3K27me3 is detected at target genes [39]. This increase might be due to the potential involvement of PRC2-MTF2. PCL is also known to contribute to the induction of high levels of H3K27me3 at PRC2 target genes [20]. Here, we investigated the interlink between MTF2 and PRC2 core subunits. Down-regulation of *Mtf2* did not affect the global level of SUZ12 or H3K27me3. However, the expression of MTF2 was partially related to the depletion of *Suz12*. At Hox B cluster genes, the binding of MTF2 was fully dependent on SUZ12. Double inhibition of MTF2 and SUZ12 re-established the level of H3K27me3 comparable to WT and induced the expression of Hox B cluster genes with deposition of active histone marker H3K4me3. Based on these observations, we speculate that remaining MTF2 in *Suz12*-KO cells might boost the enrichment of H3K27me3 at Hox genes through unexplored mechanism. PRC2 is a gigantic complex. Auxiliary subunits of this complex could also play a vital role in the assembly of H3K27me3. Recently, PRC2 has been classified into two complexes: PRC2.1 and PRC2.2. In addition to core PRC2 components (EZH2, SUZ12, EED, and RbAp46/48), PRC2.1 could be structured by the addition of one of PCL homologs (PHF1, MTF2, or PHF19) with EPOP or C10orf12. PRC2.2 could be constituted by the combination of core components together with AEBP2 and JARID2. Although these three PCL paralogs have a close evolutionary relationship, they regulate PRC2 activity in different manners [40, 41]. The role of AEBP2 is also compelling as it impedes the level of H3K27me3 at PRC2 target genes. The loss of AEBP2 can reduce the association of JARID2 with the PRC2 complex and pave the way for inclusion of MTF2 to form a hybrid complex that could not be detected in wild-type setting [41, 42]. It is possible that AEBP2 and MTF2 could compete for the regulation of H3K27me3 at PRC2 target genes. Despite different combinations of core PRC2 members with sub-stoichiometric components, it is possible that sub PRC2 complexes can control the enrichment of H3K27me3 at gene-specific levels.

**Figure 6:**
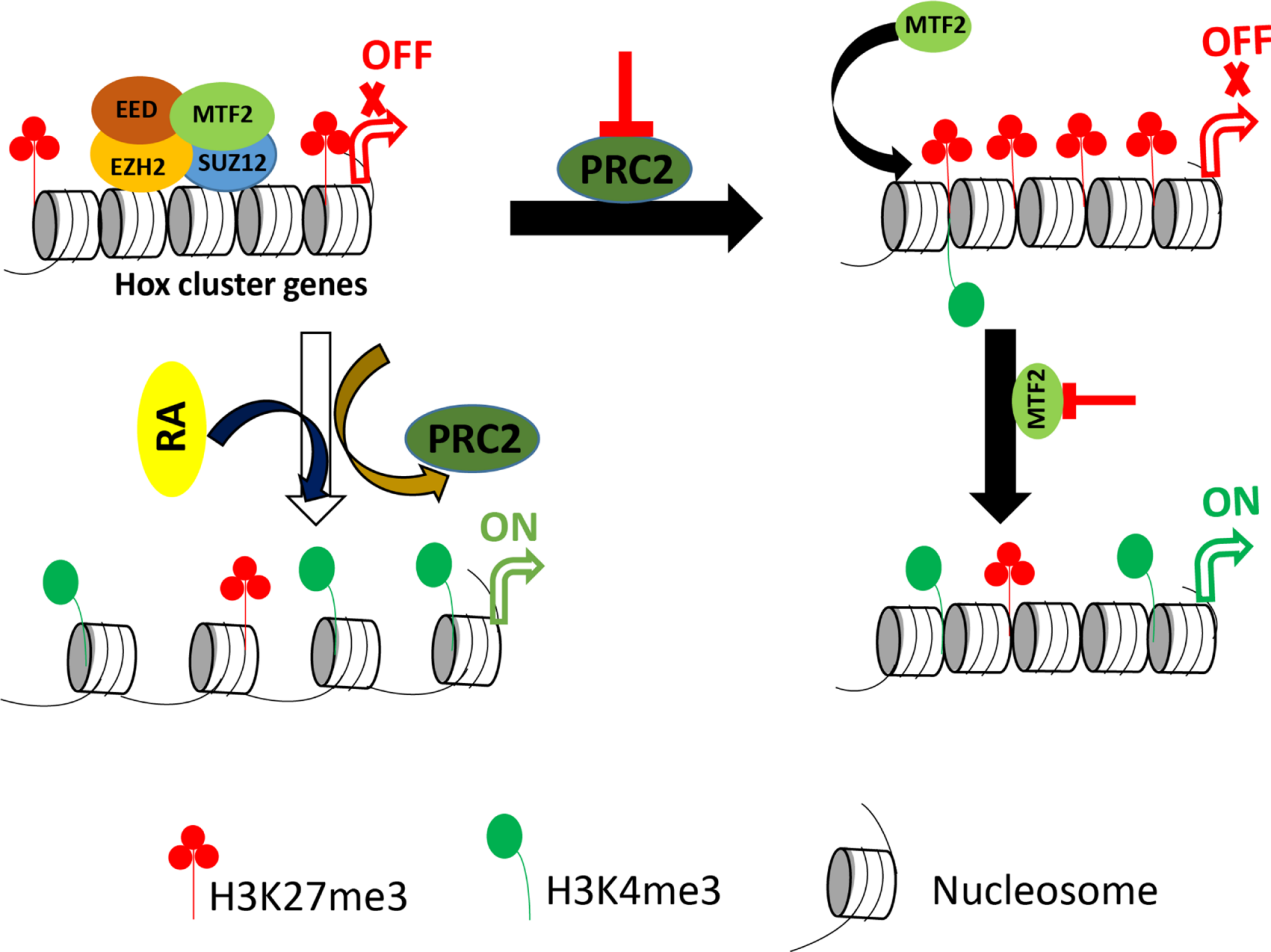
Proposed model for the regulatory role of PRC2 in undifferentiated and differentiated F9 cells Differentiation of F9 cells with RA displaces PRC2 as well as H3K27me3. It induces the transcription of Hox genes and the accumulation of H3K4me3. Conversely, the reduction of PRC2 components induces the accumulation of H3K27me3 without transcriptional induction of Hox genes. MTF2 is partially stable in the absence of SUZ12. Additional reduction of the remaining MTF2 brings back H3K27me3 with transcriptional induction of Hox genes and enrichment of H3K4me3.

Based on these observations, we could establish a relationship between PRC2 and MTF2 in F9 cells. The proposed model is shown in Figure 6. During differentiation, PRC2 and H3K27me3 are displaced, resulting in the activation of Hox genes and accumulation of H3K4me3. In undifferentiated cells, PRC2 can maintain H3K27me3 at Hox cluster genes. However, displacement of PRC2 may trigger MTF2 to accumulate H3K27me3 at target sites. Simultaneous down-regulation of *Mtf2* and *Suz12* releases the enrichment of H3K27me3 and provides a permissive environment for the introduction of H3K4me3 to activate Hox genes expression. Collectively, MTF2 associated with PRC2 might secure the repressive function of PRC2 in undifferentiated cells. But for some reason, the impairment of PRC2 reverses the chromatin state into activated form by introducing H3K4me3 at target sites. Our finding could be pertinent to identify the mechanism underlying the unusual hyper- or hypo-activity of transcription factors controlled by PRC in chromatin context.

## MATERIALS AND METHODS

### Cell culture

Mouse F9 teratocarcinoma cells were obtained from American Type Culture Collection (ATCC CRL-1720, USA) and grown using standard tissue culture protocol. Cells were cultured on 0.1% gelatin (Sigma, USA) in Dulbecco’s Modified Eagles Medium (WelGENE Inc, Korea) supplemented with 10% FBS (Fetal Bovine Serum; WelGENE Inc, Korea) and 100 µg/ml of penicillin-streptomycin (WelGENE Inc, Korea) at 37° C with 5% CO_2_ atmosphere. For differentiation, cells were treated with 1 µM retinoic acid (RA, Sigma, USA) for 72 h. Untreated cells were regarded as D0. For gene knockdown experiments, shRNA viral particles containing media were added to F9 cells and incubated at 37° C with 5% CO_2_ atmosphere for 48 h.

### Plasmids construction and generation of transgenic cells

shRNA sequences targeting *Ezh2*, *Suz12* and *Eed* (Supplementary Table 1) were designed using public TRC Portal website (https://portals.broadinstitute.org/gpp/public/seq/search). HEK293T cells were transiently transfected with pLK0.1-puro harboring shRNA, delta8.91, and pMD2.G plasmids at 3:2:1 ratio using Lipofectamine^2000^ (Invitrogen, USA). At six hours after transfection, media were changed, and viral particles were harvested after 48 h and 72 h of culture and filtered. F9 cells were transduced with shRNA viral particles for 48 h. The *suz12* knockout was performed as described previously [43] with slight modifications. Briefly, single guide RNA (sgRNA) (TCGGGCGGCAAATCCGGCGG) oligonucleotides corresponding to target protospacers were cloned in pSpCas9(BB)-2A-Puro (PX459) (Addgene, USA). Stable cell lines were selected by treating cells with puromycin for 3 days. Single KO cells were amplified for further experiments.

### Total RNA Isolation and gene expression analysis by qRT-PCR

Total RNA was isolated using TRI-solution (Bio Science, Korea) and treated with DNase I (NEB, USA). Purified RNA was reverse transcribed using Reverse transcriptase SuperScript III (Invitrogen, USA). PCR amplification was performed with the following conditions: initial denaturation at 94° C for 5 min followed by 30 cycles of 94° C for 30 sec, 60° C for 30 sec, and 72 for 30 sec. qRT-PCR was performed using SYBR Premix Ex Taq (Tli RNaseH Plus) (Takara, Japan). Primers used for RT PCR are listed in Supplementary Table 2.

### Western blot analysis

SDS-PAGE and protein transfer were performed using the standard protocol. Antibodies used in western blot are listed in Supplementary Table 3.

### Preparation of chromatin and immunoprecipitation

Chromatin immunoprecipitation (ChIP) was performed as described previously [44, 45]. ChIP-qPCR was performed using SYBR Premix Ex Taq (Takara, Japan). Antibodies used in ChIP are listed in Supplementary Table 4.

### Data analysis of ChIP-Seq and RNA-Seq

Data for ChIP-Seq and RNA-Seq were obtained using Illumina Hiseq 2500. ChIP-Seq and RNA-Seq data were analyzed as described previously [45]. Additionally, gene set enrichment assay (GSEA) [46] was performed to reveal overall expression changes for a priori defined set of genes under certain conditions. The defined set included top 500 genes near which H3K27me3 was increased upon PRC2 KD. Differentially expressed genes were identified by edgeR-3.16.5 [47] based on absolute fold change > 2 and FDR < 10^–3^. Enrichr [48] was used to investigate GO terms enriched with genes of interest. Genomic views were explored with WashU Epigenome Browser [49]. R-3.4.1 (http://www.r-project.org) was used to plot various graphs unless specifically noted. Sequencing data produced were deposited into Gene Expression Omnibus (GEO) at the National Center for Biotechnical Information (NCBI) (GSE111147).

## SUPPLEMENTARY MATERIALS FIGURES AND TABLES



## Abbreviations

EZH2: Enhancer of zeste homolog 2
SUZ12: Suppressor of zeste 12
EED: Embryonic ectoderm development
MTF2: Metal response element binding transcriptional factor 2
PRC: Polycomb repressive complex
PcG: Polycomb group
ChIP: Chromatin immunoprecipitation
GSEA: Gene set enrichment assay
ESCs: Embryonic stem cells
PCL: Polycomb like
EC: Embryonic carcinoma
RA: Retinoic acid
shRNA: short hairpin RNA
GO: Gene ontology
FDR: False discovery rate
GEO: Gene expression omnibus
