# Supplementary material for: A novel role of metal response element binding transcription factor 2 at the Hox gene cluster in the regulation of H3K27me3 by polycomb repressive complex 2
Source: Oncotarget. 2018 May 29;9(41):26572–85. doi: 10.18632/oncotarget.25505 (PMC5995182; doi:10.18632/oncotarget.25505)
Supplement: Supplementary file 1 [file oncotarget-09-26572-s001.pdf]

A novel role of metal response element binding transcription factor 2 at the Hox gene cluster in the regulation of H3K27me3 by polycomb repressive complex 2

SUPPLEMENTARY MATERIALS

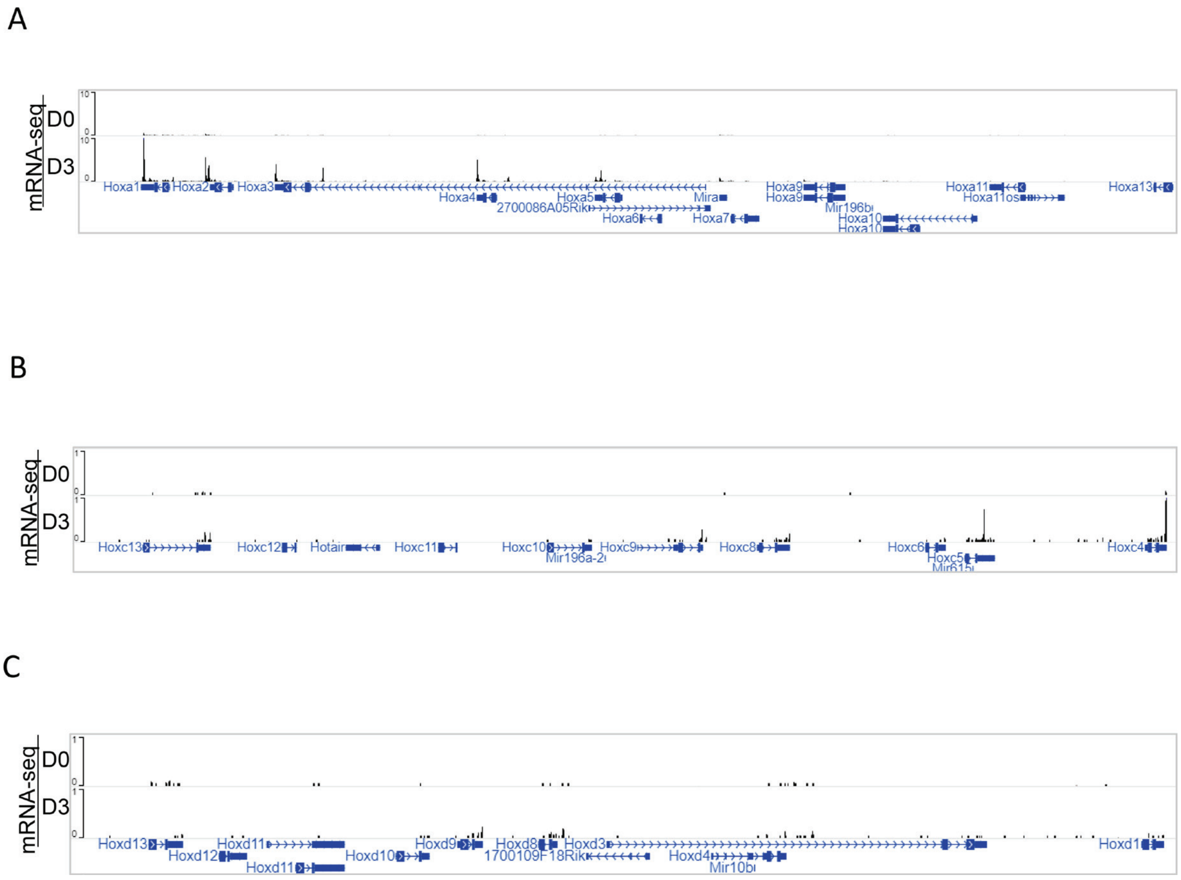

Supplementary Figure 1: Hox genes expression in RA differentiating F9 cells. Genome browser view of Hox A (**A**), Hox C (**B**), and Hox D (**C**) cluster genes expression in differentiating F9 cells.

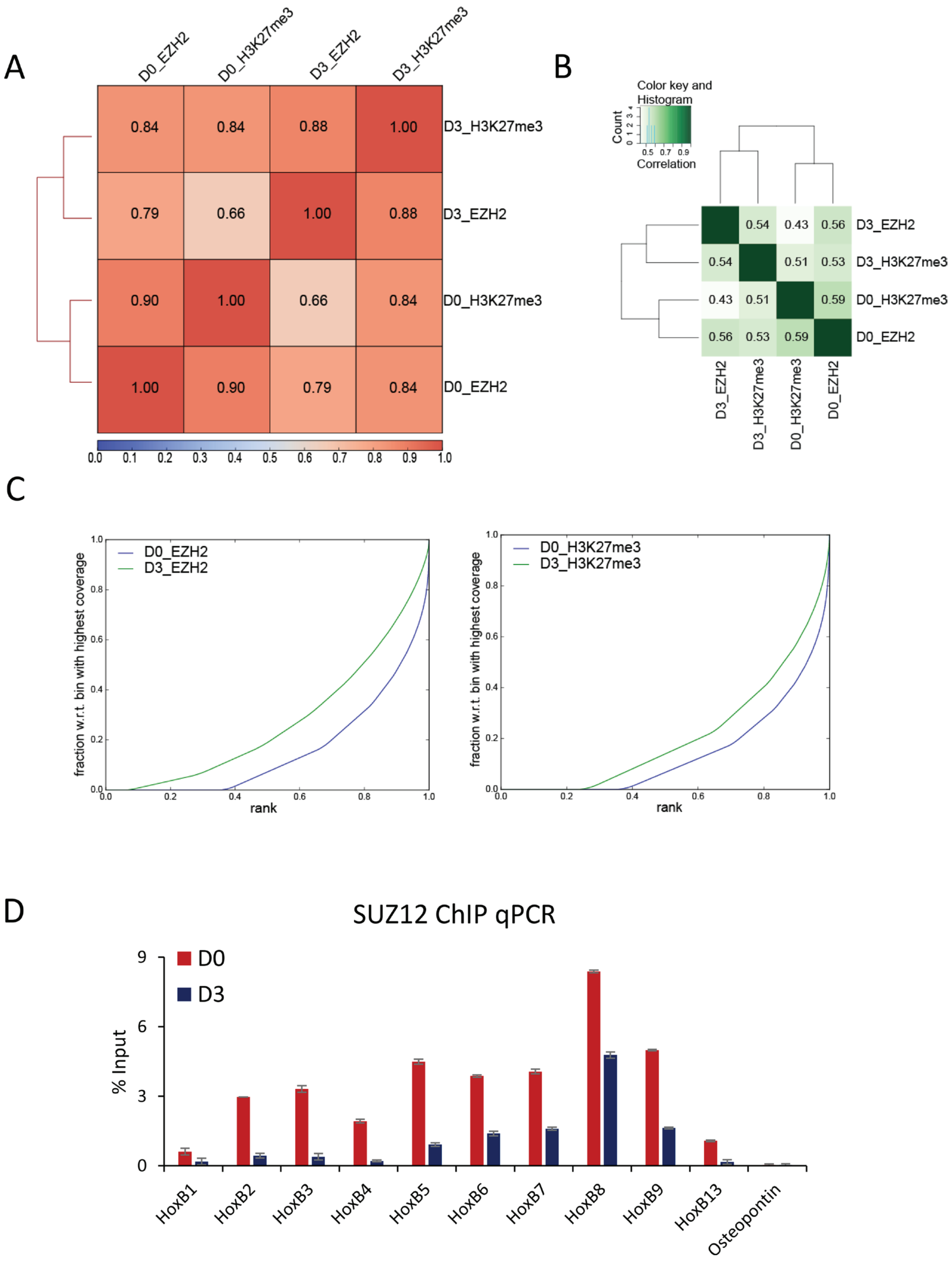
A
D0_EZH2
D0_H3K27me3
D3_EZH2
D3_H3K27me3
0.84 0.84 0.88 1.00 D3_H3K27me3
0.79 0.66 1.00 0.88 D3_EZH2
0.90 1.00 0.66 0.84 D0_H3K27me3
1.00 0.90 0.79 0.84 D0_EZH2
0.0 0.1 0.2 0.3 0.4 0.5 0.6 0.7 0.8 0.9 1.0
B
Color key and Histogram
Count
Correlation
0.54 0.43 0.56 D3_EZH2
0.54 0.51 0.53 D3_H3K27me3
0.43 0.51 0.59 D0_H3K27me3
0.56 0.53 0.59 D0_EZH2
D3_EZH2
D3_H3K27me3
D0_H3K27me3
D0_EZH2
C
D0_EZH2
D3_EZH2
fraction w.r.t. bin with highest coverage
rank
D0_H3K27me3
D3_H3K27me3
fraction w.r.t. bin with highest coverage
rank
D
SUZ12 ChIP qPCR
D0
D3
% Input
9
6
3
0
HoxB1
HoxB2
HoxB3
HoxB4
HoxB5
HoxB6
HoxB7
HoxB8
HoxB9
HoxB13
Osteopontin

E

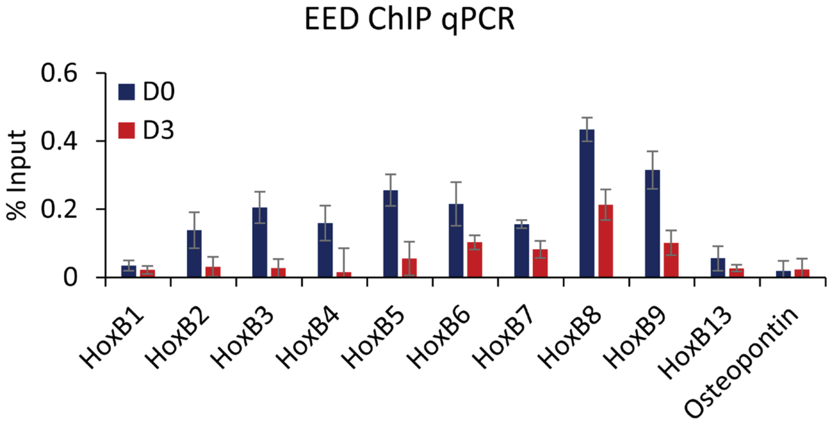
EED ChIP qPCR
% Input
0.6
0.4
0.2
0
D0
D3
HoxB1
HoxB2
HoxB3
HoxB4
HoxB5
HoxB6
HoxB7
HoxB8
HoxB9
HoxB13
Osteopontin

F

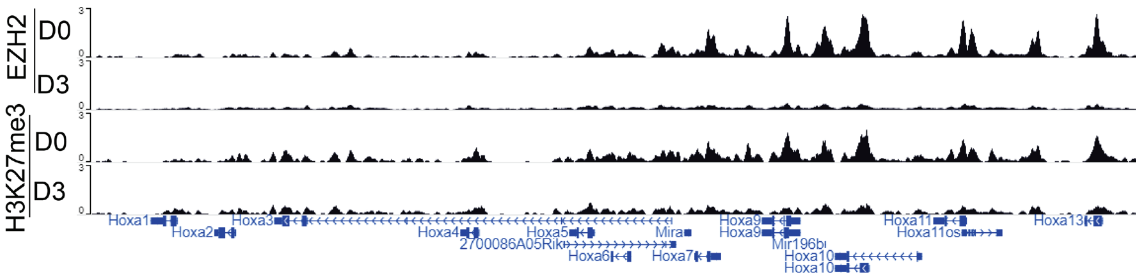
EZH2
D0
D3
H3K27me3
D0
D3
Hoxa1
Hoxa2
Hoxa3
Hoxa4
2700086A05Rik
Hoxa5
Hoxa6
Hoxa7
Mira
Hoxa9
Hoxa9
Mir196b
Hoxa10
Hoxa10
Hoxa11
Hoxa11os
Hoxa13

G

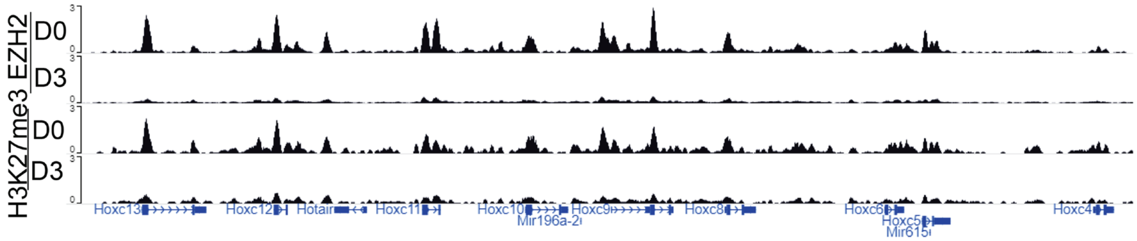
EZH2
D0
D3
H3K27me3
D0
D3
Hoxc13
Hoxc12
Hotair
Hoxc11
Hoxc10
Mir196a-2
Hoxc9
Hoxc8
Hoxc6
Hoxc5
Mir615
Hoxc4

H

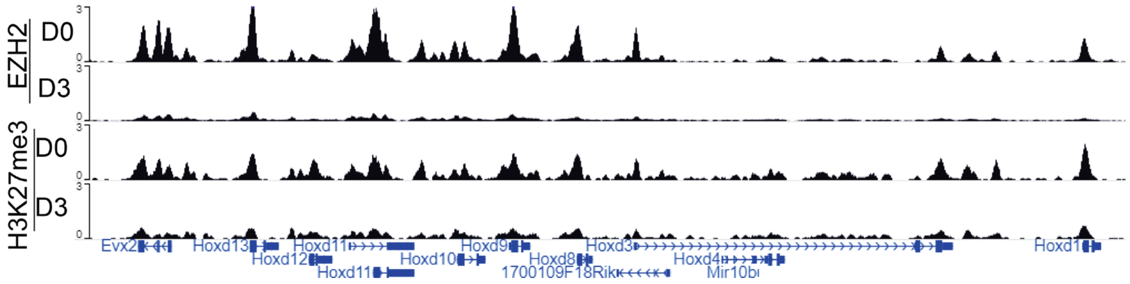
EZH2
D0
D3
H3K27me3
D0
D3
Evx2
Hoxd13
Hoxd12
Hoxd11
Hoxd11
Hoxd10
Hoxd9
Hoxd8
1700109F18Rik
Hoxd3
Hoxd4
Mir10b
Hoxd1

I

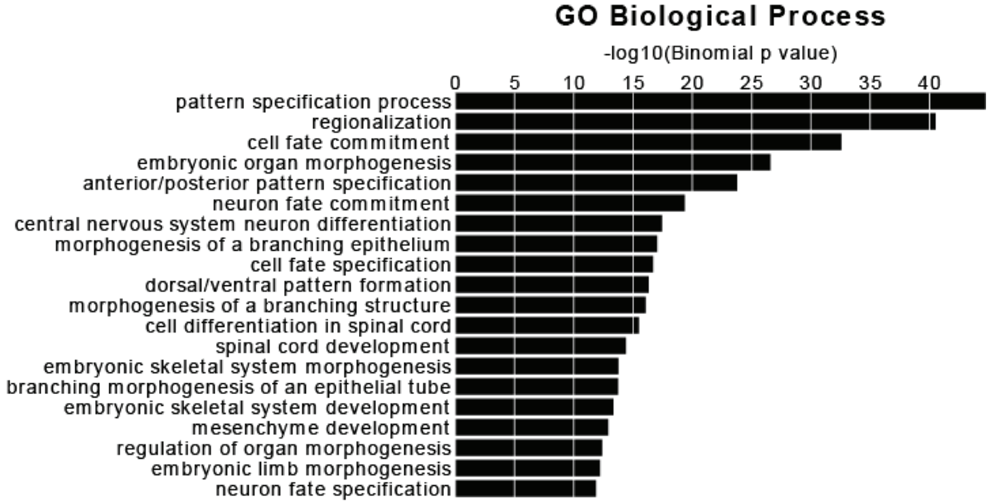

J

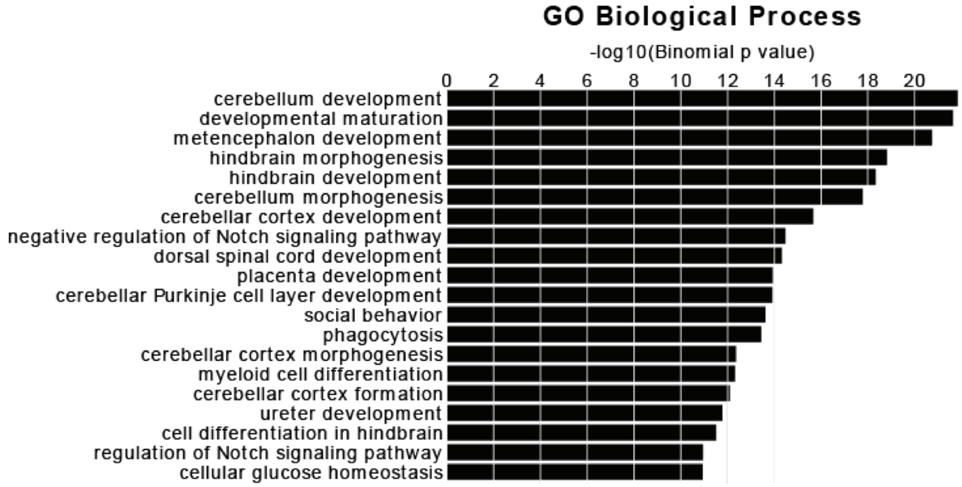

Supplementary Figure 2: Displacement of PRC2 and H3K27me3 from target genes during cellular differentiation. (**A**) Correlation coefficients between EZH2 and H3K27me3 at D0 and D3 demonstrate strong correlation with each other in whole genome level and (**B**) at localized peaks level. (**C**) Finger point plot shows strong enrichment of EZH2 at D0 which is significantly decreased with RA differentiation (left). H3K27me3 did not show distinct decrease in enrichment at D3 compared to that at D0 (right). (**D**) ChIP qPCR assay for the confirmation of SUZ12 and (**E**) EED removal from Hox B loci in differentiating F9 cells. Error bars represent mean ± SD of three biological replicates. $^{*}P < 0.05$, $^{**}P < 0.01$, $^{***}P < 0.001$ by Student's two-tailed *t*-test. For all bars, *P* values were less than 0.001 unless otherwise specified. Genome browser view of EZH2 and H3K27me3 ChIP-Seq at D0 and D3 of Hox A loci (**F**), Hox C loci (**G**), and Hox D loci (**H**). (**I**) GO analysis of EZH2 and H3K27me3 co-bound genes. Bars represent $-\log_{10}$ of *p*-values. (**J**) GO analysis of H3K27me3 bound genes. Bars represent $-\log_{10}$ of *p*-values.

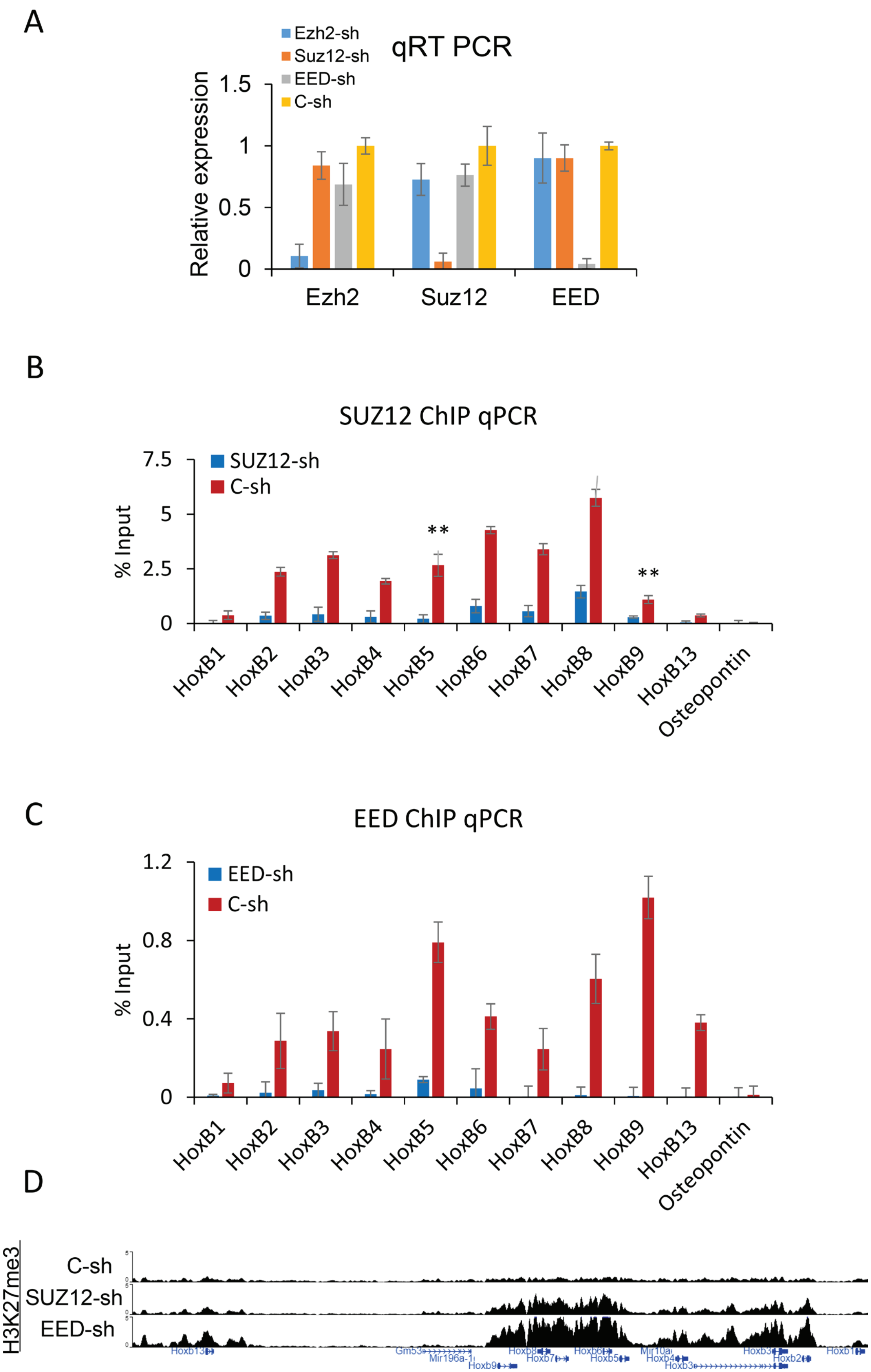
A
qRT PCR
Ezh2-sh
Suz12-sh
EED-sh
C-sh
Relative expression
1.5
1
0.5
0
Ezh2
Suz12
EED
B
SUZ12 ChIP qPCR
SUZ12-sh
C-sh
% Input
7.5
5
2.5
0
**
**
HoxB1
HoxB2
HoxB3
HoxB4
HoxB5
HoxB6
HoxB7
HoxB8
HoxB9
HoxB13
Osteopontin
C
EED ChIP qPCR
EED-sh
C-sh
% Input
1.2
0.8
0.4
0
HoxB1
HoxB2
HoxB3
HoxB4
HoxB5
HoxB6
HoxB7
HoxB8
HoxB9
HoxB13
Osteopontin
D
H3K27me3
C-sh
SUZ12-sh
EED-sh
Hoxb13
Hoxb9
Hoxb8
Hoxb7
Hoxb6
Hoxb5
Hoxb4
Hoxb3
Hoxb2
Hoxb1

E

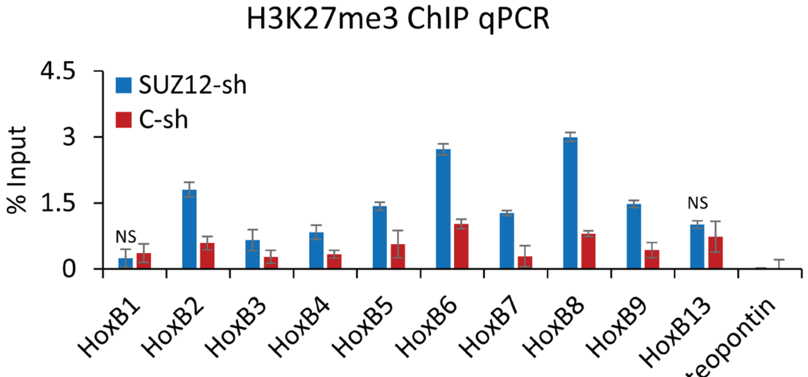
H3K27me3 ChIP qPCR
% Input
4.5
3
1.5
0
SUZ12-sh
C-sh
NS
NS
HoxB1
HoxB2
HoxB3
HoxB4
HoxB5
HoxB6
HoxB7
HoxB8
HoxB9
HoxB13
Osteopontin

F

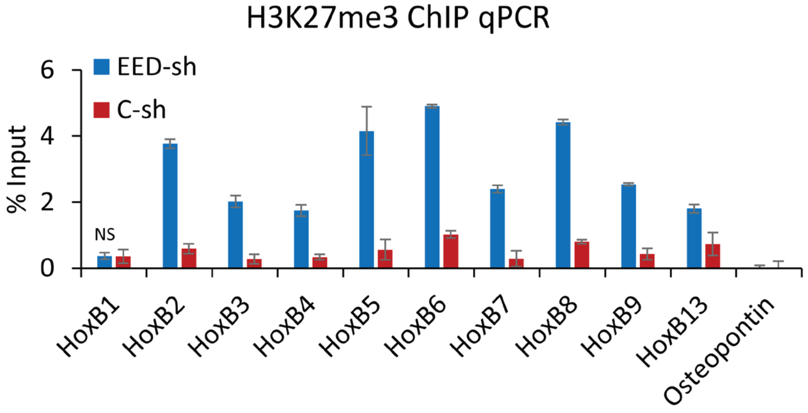
H3K27me3 ChIP qPCR
% Input
6
4
2
0
EED-sh
C-sh
NS
HoxB1
HoxB2
HoxB3
HoxB4
HoxB5
HoxB6
HoxB7
HoxB8
HoxB9
HoxB13
Osteopontin

G

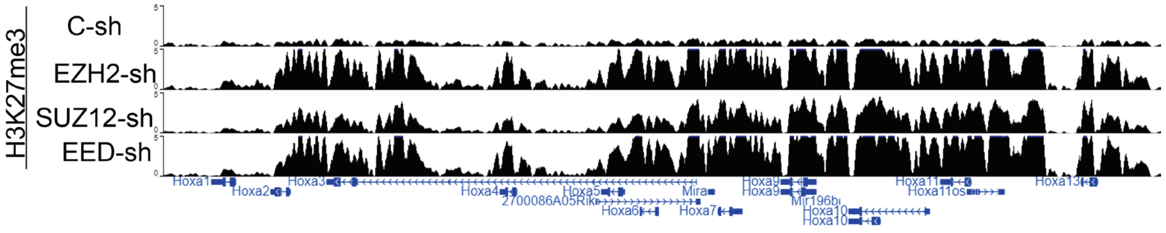
H3K27me3
C-sh
EZH2-sh
SUZ12-sh
EED-sh
Hoxa1
Hoxa2
Hoxa3
Hoxa4
2700086A05Rik
Hoxa5
Hoxa6
Mira
Hoxa7
Hoxa9
Hoxa9
Mir196b
Hoxa10
Hoxa10
Hoxa11
Hoxa11os
Hoxa13

H

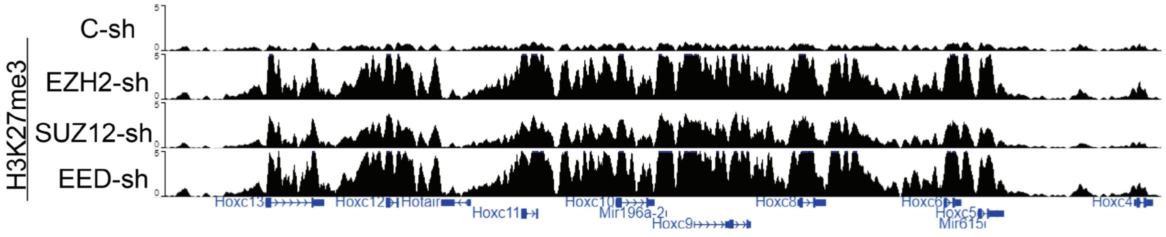
H3K27me3
C-sh
EZH2-sh
SUZ12-sh
EED-sh
Hoxc13
Hoxc12
Hotair
Hoxc11
Hoxc10
Mir196a-2
Hoxc9
Hoxc8
Hoxc6
Hoxc5
Mir615
Hoxc4

I

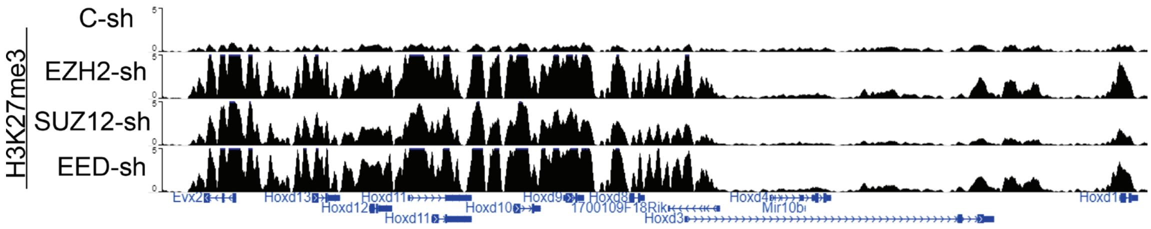
C-sh
EZH2-sh
SUZ12-sh
EED-sh
H3K27me3
Evx2
Hoxd13
Hoxd12
Hoxd11
Hoxd11
Hoxd10
Hoxd9
Hoxd8
1700109F18Rik
Hoxd4
Mir10b
Hoxd3
Hoxd1

J

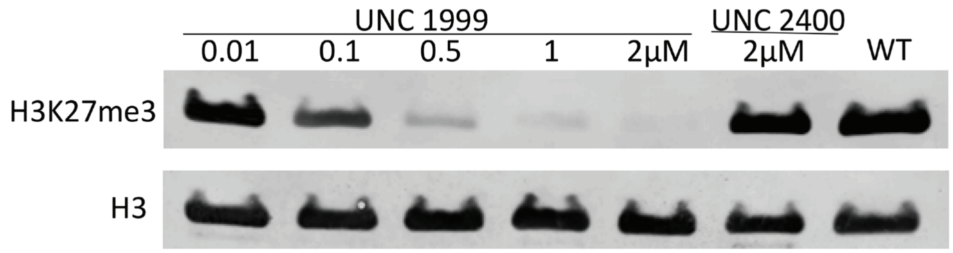
UNC 1999
0.01
0.1
0.5
1
2μM
UNC 2400
2μM
WT
H3K27me3
H3

K

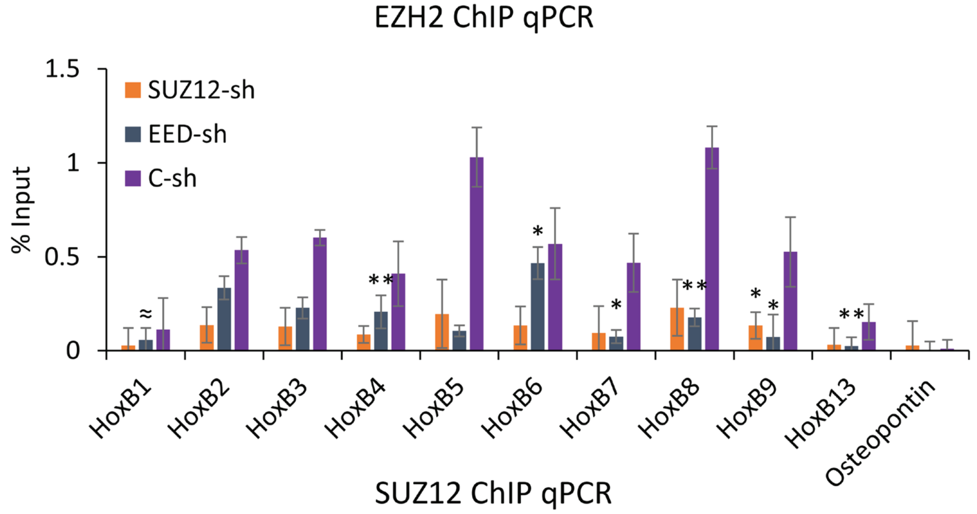
EZH2 ChIP qPCR
% Input
1.5
1
0.5
0
SUZ12-sh
EED-sh
C-sh
HoxB1
HoxB2
HoxB3
HoxB4
HoxB5
HoxB6
HoxB7
HoxB8
HoxB9
HoxB13
Osteopontin

L

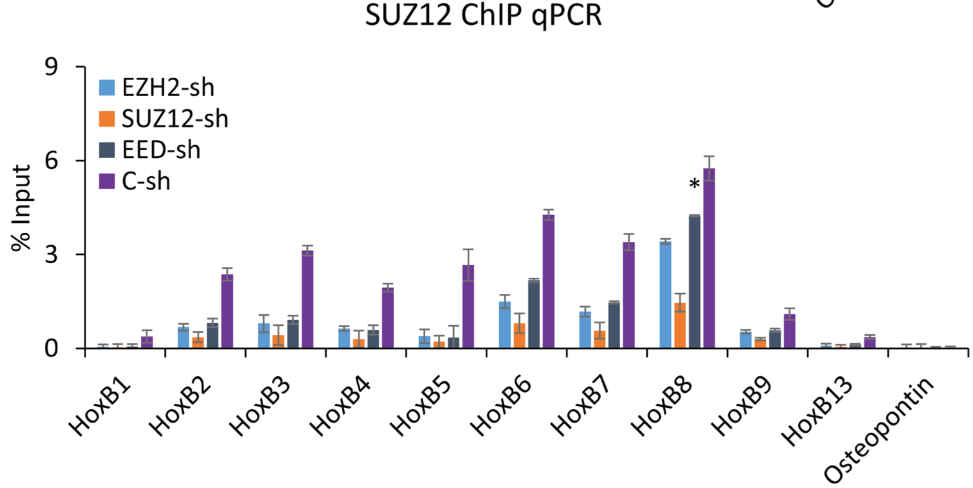
SUZ12 ChIP qPCR
% Input
9
6
3
0
EZH2-sh
SUZ12-sh
EED-sh
C-sh
HoxB1
HoxB2
HoxB3
HoxB4
HoxB5
HoxB6
HoxB7
HoxB8
HoxB9
HoxB13
Osteopontin

M

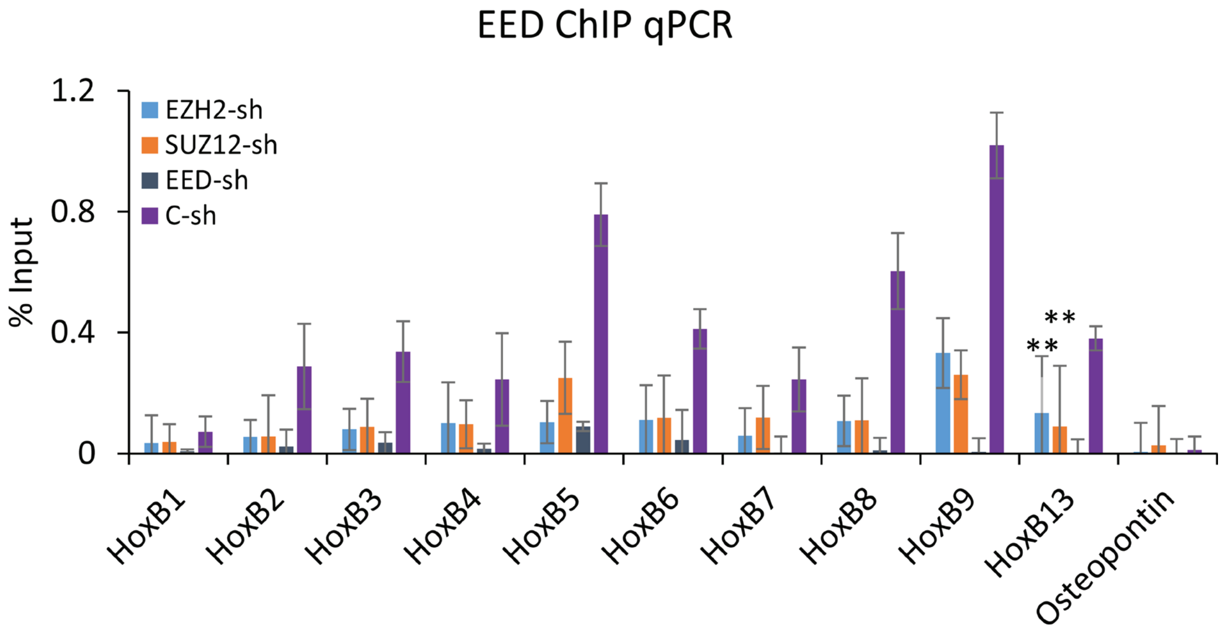

Supplementary Figure 3: Effect of PRC2 knockdown on H3K27me3 deposition. (**A**) qRT PCR analysis for knockdown confirmation of *Ezh2*, *Suz12*, and *Eed*. Error bars represent mean ± SD of three biological replicates. (**B**) ChIP qPCR assay for *SUZ12* binding to Hox B loci after shRNA depletion. *Osteopontin* was used as negative control region. Error bars represent mean ± SD of three biological replicates. $^{*}P < 0.05$, $^{**}P < 0.01$, $^{***}P < 0.001$ by Student's two-tailed *t*-test. For all bars, *P* values were less than 0.001 unless otherwise specified. (**C**) ChIP qPCR assay for EED binding to Hox B loci after shRNA knockdown. Osteopontin was used as negative control region. Error bars represent mean ± SD of three biological replicates. $^{*}P < 0.05$, $^{**}P < 0.01$, $^{***}P < 0.001$ by Student's two-tailed *t*-test. For all bars, *P* values were less than 0.001 unless otherwise specified. (**D**) Genome browser view comparing H3K27me3 increase at Hox B loci in *Suz12* and *Eed* knockdown compared to C-sh. (**E**) ChIP qPCR to confirm the increase of H3K27me3 in *Suz12* knockdown and (**F**) *Eed* knockdown of Hox B cluster genes. NS: Not Significant. *Osteopontin* was used as negative control region. Error bars represent mean ± SD of three biological replicates. $^{*}P < 0.05$, $^{**}P < 0.01$, $^{***}P < 0.001$ by Student's two-tailed *t*-test. For all bars, *P* values were less than 0.001 unless otherwise specified. Genome browser view of Hox A (**G**), Hox C (**H**), and Hox D (**I**) loci showing increase in H3K27me3 deposition in *Ezh2*, *Suz12*, and *Eed* knocked down compare to control. (**J**) Western blot assay showing decrease in global H3K27me3 protein level after treatment with EZH1/2 inhibitor compare to UNC2400 control and WT. H3 was used as a loading control. (**K**) ChIP qPCR showing decrease in binding of EZH2 in *Suz12*-sh and *Eed*-sh compared to C-sh. *Osteopontin* was used as a negative control. (**L**) ChIP qPCR showing decrease in the binding of SUZ12 in *Ezh2*-sh, *Suz12*-sh, and *Eed*-sh compared to C-sh. Osteopontin was used as a negative control. (**M**) ChIP qPCR showing decrease in the binding of EED in *Ezh2*-sh, *Suz12*-sh, and *Eed*-sh compared to C-sh. Osteopontin was used as a negative control. Error bars represent mean ± SD of three biological replicates. $^{*}P < 0.05$, $^{**}P < 0.01$, $^{***}P < 0.001$ by Student's two-tailed *t*-test. For all bars, *P* values were less than 0.001 unless otherwise specified.

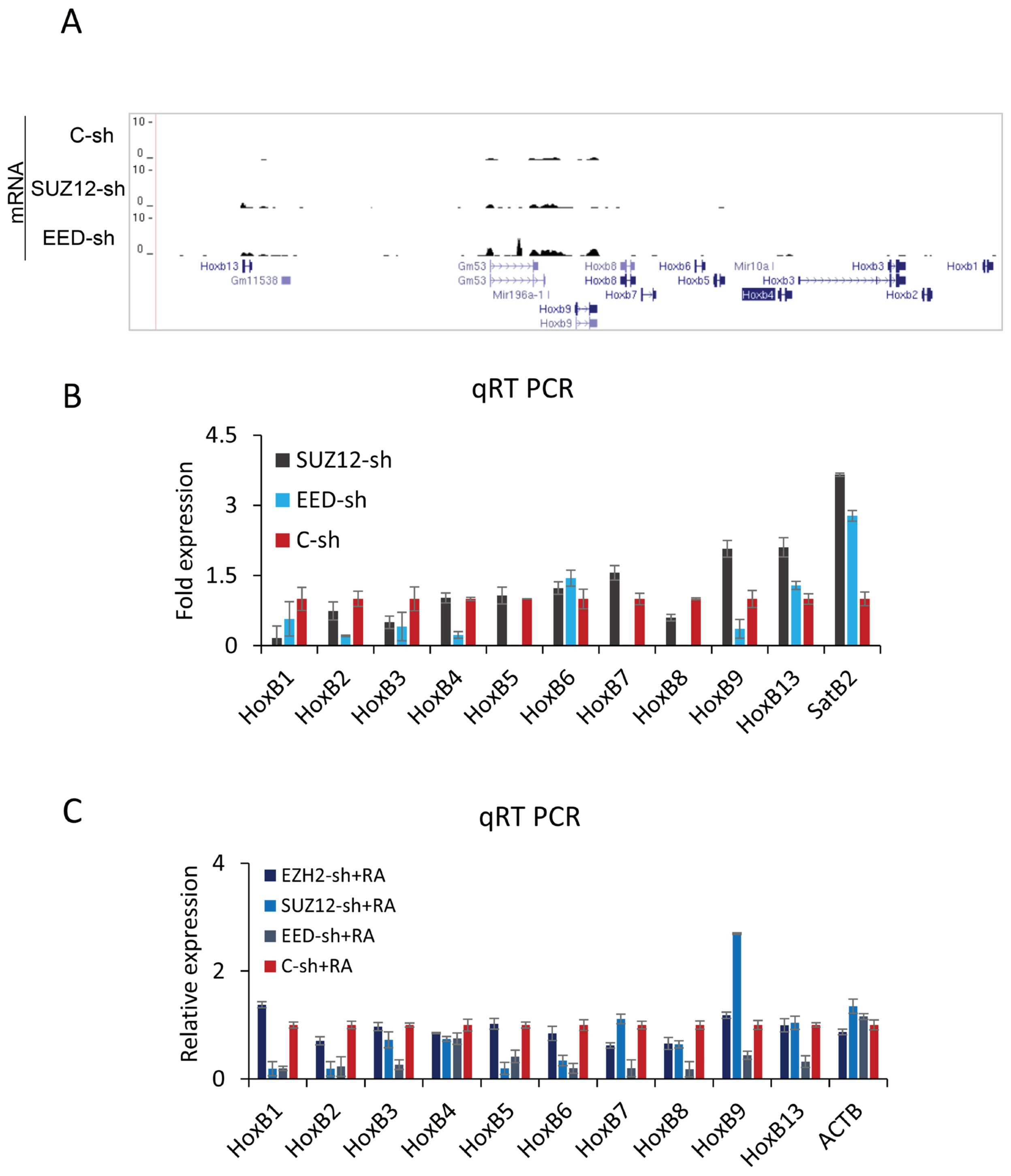

A
mRNA
C-sh
SUZ12-sh
EED-sh
Hoxb13
Gm11538
Gm53
Mir196a-1
Hoxb9
Hoxb8
Hoxb7
Hoxb6
Hoxb5
Mir10a
Hoxb4
Hoxb3
Hoxb2
Hoxb1
B
qRT PCR
Fold expression
SUZ12-sh
EED-sh
C-sh
HoxB1
HoxB2
HoxB3
HoxB4
HoxB5
HoxB6
HoxB7
HoxB8
HoxB9
HoxB13
SatB2
C
qRT PCR
Relative expression
EZH2-sh+RA
SUZ12-sh+RA
EED-sh+RA
C-sh+RA
HoxB1
HoxB2
HoxB3
HoxB4
HoxB5
HoxB6
HoxB7
HoxB8
HoxB9
HoxB13
ACTB

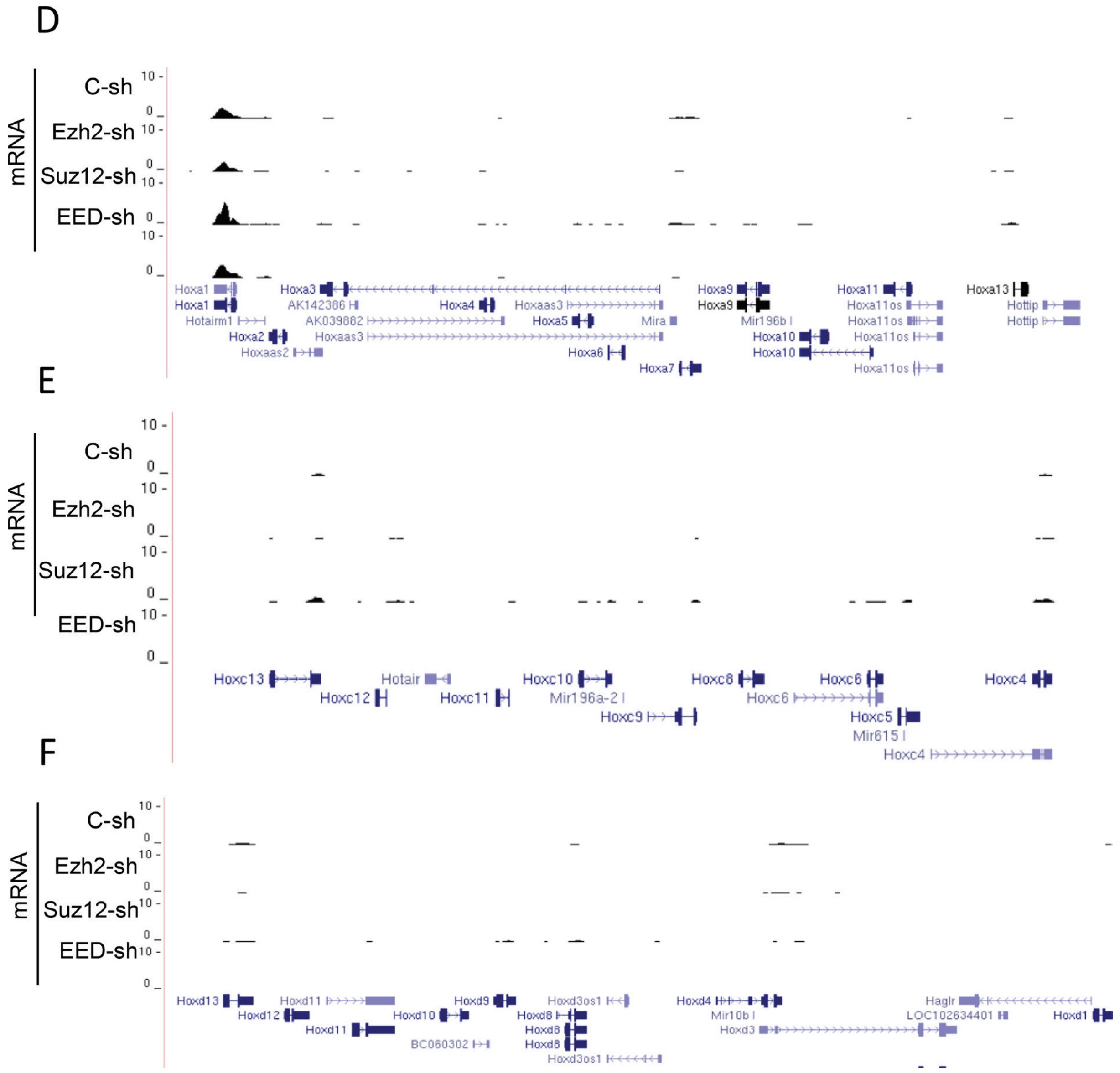

Supplementary Figure 4: Effect of PRC2 knockdown on Hox genes expression. (**A**) Genome browser view of Hox B loci showing no changes in transcription in *Suz12* or *Eed* knockdown compare to control knockdown. (**B**) qRT PCR assay for the confirmation mRNA-Seq. *SatB2* was used as a positive control. Error bars represent mean ± SD of three biological replicates. (**C**) qRT PCR assay showing decrease in expression of Hox B cluster genes after PRC2 knockdown and RA treatment. Error bars represent mean ± SD of three biological replicates. Genome browser view of Hox A (**D**), Hox C (**E**), and Hox D (**F**) loci showing no genes expression.

A

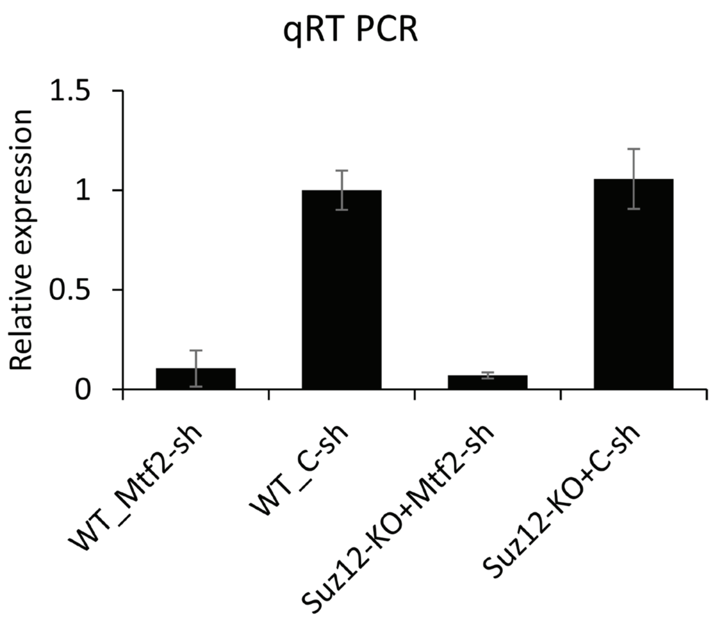

B

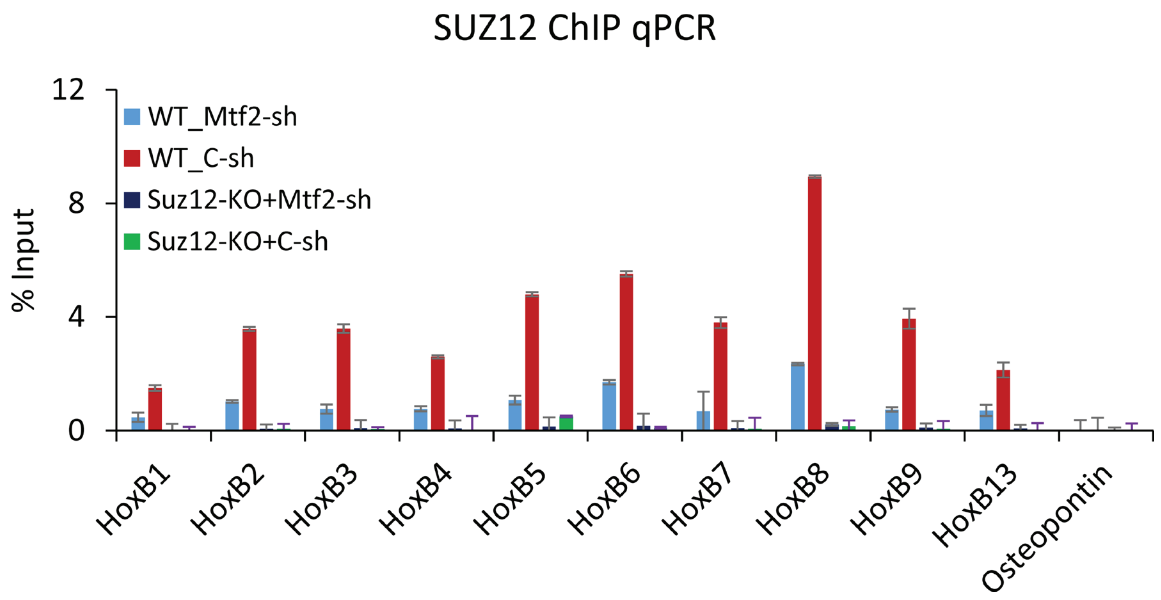

Supplementary Figure 5: Role of MTF2 in PRC2 depleted cells. (**A**) Confirmation of *Mtf2* knockdown by qRT PCR in WT and *Suz12*-KO. Error bars represent mean ± SD of three biological replicates. (**B**) The binding of SUZ12 was significantly decreased with knockdown of *Mtf2* in WT and strongly decreased in *Suz12*-KO+*Mtf2*-sh and *Suz12*-KO+C-sh cells. Osteopontin was used as a negative control. Error bars represent mean ± SD of three biological replicates. $^{*}P < 0.05$, $^{**}P < 0.01$, $^{***}P < 0.001$ by Student's two-tailed *t*-test. For all bars, *P* values were less than 0.001 unless otherwise specified.

Supplementary Table 1: List of shRNA sequences used for knockdown

Gene name	shRNA sequence
Ezh2	CCGG GCACAAGTCATCCCGTTAAAG CTCGAG CTTTAACGG GATGACTT GTGCTTTTTG
Suz12	CCGGGCTGACAATCAAATGAATCATCTCGAGATGATTCATTTGATTGTCAGCTTTTTG
EED	CCGGTCTTGCTAGTAAGGGCACATACTCGAGTATGTGCCCTTACTAGCAAGATTTTTG
Mtf2#1	CCGGGGCCCTGGAGACTGGTATTTACTCGAGTAAATACCAGTCTCCAGGGCCTTTTTG
Mtf2#3	CCGGGCATGTTCTGGAGGCATTAAACTCGAGTTTAATGCCTCCAGAACATGCTTTTTG
control	CCGGCCGCAGGTATGCACGCGTCTCGAGACGCGTGCATACCTGCGGTTTTTG

Supplementary Table 2: List of RT PCR primers

Gene name	Forward primer	Reverse primer
HoxB1	GAACTGAGCAGACCGCAACC	TCCTTCTCCAGCTCCGTCAG
HoxB2	CTACCGGACCTCAATTTCTTCG	TAGGGAAACTGCAAGTCGATGG
HoxB3	CTCAGCGTGTTCTCTGCAGTCC	CCCGTTATTGCTGTTGCTAGTGG
HoxB4	CTCTCGGACCGCCTACACTC	CCGAGCGGATCTTGGTGTT
HoxB5	ATTCCCCTGGATGAGGAAGC	CACTTCATGCGACGGTTCTG
HoxB6	TGTTCGGAGAGACCGAGGAG	GAGCTGAGACGCACTGAGCA
HoxB7	GCTCGAACCGAGTTCCTTCA	GGGTCTGGTAGCGCGTGTAG
HoxB8	CGGCAACTTCTACGGCTACG	CGTGCGATACCTCGATCCTC
HoxB9	GAGCTGGCTACGGGGACAAT	GGAGTCTGGCCACTTCATGC
HoxB13	CTCGCCACGATTCTCTGCTT	TGCATACTCCCGCTCCAACT
SatB2	GATCGCATTTACCAGGATGAGC	CTCGTCATAAATGGCAGCTGTG
Ezh2	CATGTGCAGCTTTCTGTTCAA	GGATTTCCATTTCTCGTTCG
Suz12	GGGGAATATGAAGTGGCCATG	GCAGGTTTAACAGAACCAGGCTT
EED	CTGGGGAAAGGGAAAATGGA	GGGTGGCTGGTGTTGCTATC
Mtf2	GCACACCTATGCCTTTACAACCTAAG	TGCCTCCAGAACATGCTCATATC
18srRNA	CATTCGAACGTCTGCCCTATC	CCTGCTGCCTTCCTTGGA
ACTB	GCATTGTTACCAACTGGGACG	CCAGAGGCATACAGGGACAG
Enox1	CATACATGCAGCAGCTGGACTC	TCAAAGGCACACAACTTCCATC
Bmp4	CAGCTTCTCTGAGCCTTTCCAG	ACCATCAGCATTCGGTTACCAG
Dab1	CCTACATTGCGAAGGACATCACA	CAAGTCCAGGATAACAGGTTCAGC
Rxrg	CCTCTGAGGTGGAGACTCTTCG	GAGGTGTTCCAGGCATTTCAAG
GATA4	CAGCAGCAGCAGTGAAGAGATG	ACAGCTTCAGAGCAGACAGCAC
GATA6	GCAGTGGCTCTGTCCCTATGAC	TTTCTCCCACTGCAGACATCAC
Sox17	CTTTATGGTGTGGGCCAAAGAC	CGCTTCTCTGCCAAGGTCAAC
Dab2	CTCAGCCTGCATCTTCTGATCC	CAACATGTTTCTGGCTGTCTGC
GAPDH	GGACTCCTATGTGGGTGACGAG	GGTACGACCAGAGGCATACAGG

Supplementary Table 3: List of antibodies used for ChIP and western blot

Target	Cat#	Manufacturer	Applications
EZH2	Ab3748	Abcam	ChIP/WB
EZH2	39875	Active Motif	ChIP
SUZ12	3737	Cell signalling	ChIP
SUZ12	sc-46264	Santa Cruz	WB
EED	09-774	Millipore	ChIP
EED	ab4469	Abcam	WB
GAPDH	sc-25778	Santa Cruz	WB
H3K27me3	07-449	Millipore	ChIP/WB
H3K4me3	Ab8580	Millipore	ChIP
MTF2	16208-1-AP	Proteintech	ChIP/WB
H3	06-755	Millipore	WB
IgG	sc-2027	Santa Cruz	ChIP
IgG	Ab46540	Abcam	ChIP

Supplementary Table 4: List of ChIP primers

**PRC2 and H3K27me3 ChIP primers**		
**Gene name**	**Forward primer**	**Reverse primer**
HoxB1	TCAGCCTACGACCTCCTCTCTG	AAGTGGCTCCTCTGAGCCCTAC
HoxB2	CGAGTTCCCCTGGATGAAAGAG	TTTACCCACCTGATGGTGATCC
HoxB3	AAGAAAGACCAGAAGGCCAAGG	GGTCATGGAGTGTAAGGCGTTC
HoxB4	GTCGGCCAACCACTTAAAACC	CCCTGCCCTACCTAATCTCCAC
HoxB5	TGAAGAAATAGAGGCTGGATGC	GCAACGTTATTCCGGTTAAAGG
HoxB6	GGGAAAGACTACTCCCGACAGG	AGGGTCTGGTAGCGTGTGTAGG
HoxB7	TGGATGCGAAGCTCAGGTAAAG	CGGGAATAACCTGGACACAGAG
HoxB8	TCGCAAATCCAGGAGTTCTACC	AGTCTGCGTACTGCACCAGGTC
HoxB9	TGTCCATTTCTGGGACGCTTAG	AAGCTGCACGAGGGGAAGTC
HoxB13	TTGGAGGCGGGTACTACTCTTG	GTAGCCCGGATAGAAGGCAAAC
Shlf2	GGGACTGGAGGGACTCTAAAGG	TTGGGCAGACTGTAACACAAGC
Shbg	CTCCTTCTTGCACCTGGATAGG	AAGAGAGACTCTGTGGCCTTGC
Osteopontin	GATCAAGCCATAGCCCTTCA	TCACCATTCGGATGAGTCTG
**H3K4me3 ChIP primers**		
HoxB1	TCAGCCTACGACCTCCTCTCTG	AAGTGGCTCCTCTGAGCCCTAC
HoxB2	TGCAGACTCCTGTCTCCAGATG	TAGGGAAACTGCAAGTCGATGG
HoxB3	ATCCTCTCTCCTGGGGTTTCTC	GAGACAGTGGCTCTGGGCTTAC
HoxB4	CGCTTTACAGTGAAGGGACCTC	GGCGATGTAATTATGTGGCTCTG
HoxB5	AGTCCCTGCCCTGCACTAAC	GCGCTGGCCTCGTCTATTTC
HoxB6	AAGTCCCAGCTGCACAGTAAGG	TCAGATCCGGACAAGAGCTAGG
HoxB7	ACGTCCCCGACTACAAATCATC	CAAAGGCGCAAGAAGTTTGTTC
HoxB8	CACAGCTCCCTACCAGCAGAAC	AGTCTGCGTACTGCACCAGGTC
HoxB9	TGTCCATTTCTGGGACGCTTAG	AAGCTGCACGAGGGGAAGTC
HoxB13	AAAGAGACAGGCGGAGAGGAAG	GTCTTGACGCAAGAGACGCAAG
Osteopontin	GATCAAGCCATAGCCCTTCA	TCACCATTCGGATGAGTCTG
